# Importin α1 contributes to Venezuelan equine encephalitis virus-induced cell death, but is not required for the capsid-mediated blockage of nucleocytoplasmic trafficking

**DOI:** 10.3389/fmicb.2026.1784611

**Published:** 2026-03-25

**Authors:** Abdullahi T. Jamiu, Ivan Akhrymuk, Grace Boyer, Gregory M. Petruncio, Maryna Akhrymuk, Bryan M. Delfing, Hui-Chen Foreman, Kenneth W. Foreman, Dmitri K. Klimov, Mikell Paige, Kylene Kehn-Hall

**Affiliations:** 1 Department of Biomedical Sciences and Pathobiology, Virginia-Maryland College of Veterinary Medicine, Virginia Polytechnic Institute and State University, Blacksburg, VA, United States; 2 Center for Emerging, Zoonotic, and Arthropod-Borne Pathogens (CeZAP), Virginia Polytechnic Institute and State University, Blacksburg, VA, United States; 3 Department of Chemistry and Biochemistry, George Mason University, Manassas, VA, United States; 4 School of Systems Biology, George Mason University, Manassas, VA, United States; 5 Mercy LLC, Roswell, GA, United States

**Keywords:** alphavirus, capsid, importin alpha, nuclear-cytoplasmic trafficking, Venezuelan equine encephalitis virus

## Abstract

Alphaviruses are single-stranded, enveloped, positive-sense RNA viruses within the family *Togaviridae* that cause significant human and veterinary disease worldwide. Venezuelan equine encephalitis virus (VEEV) is regarded as a prototypic encephalitic alphavirus, serving as a valuable model to study neuroinvasive alphaviruses, for which no FDA-approved therapeutics or vaccines are currently available. Targeting host factors required for viral pathogenesis represents a promising antiviral strategy with a reduced likelihood of resistance development. Nuclear transport receptors, including importins, are exploited by viruses to support infection and evade host antiviral responses. In addition to its structural role in virion assembly, VEEV capsid is a key virulence determinant that blocks host nucleocytoplasmic trafficking and mediates cytopathogenicity. Although the capsid protein is known to utilize the importin α/β1 pathway, the specific importin α isoform involved and its functional relevance remain unclear. Here, the role of importin α1 (KPNA2) in VEEV capsid subcellular localization, nucleocytoplasmic trafficking blockage, viral replication, and virus-induced cell death using complementary genetic and pharmacological approaches was investigated. Using recombinant VEEV expressing a V5-tagged capsid, full-length capsid was shown to associate with importin α1 and CRM1 in infected cells. Genetic ablation or pharmacological disruption (with compounds I2 or 1564) of importin α1 significantly reduced capsid nuclear localization, which was reversed following KPNA2 complementation, identifying importin α1 as a key mediator of capsid nuclear import. However, the loss or inhibition of importin α1 was insufficient to relieve capsid-inhibition of nucleocytoplasmic trafficking, suggesting functional redundancy as we found that the capsid also interacts with both importin α4 and importin α3. Importantly, loss or inhibition of importin α1 function partly protected cells from VEEV-induced cell death. While viral replication was unaffected by importin α1 loss, treatment with compounds 1564 or I2 reduced VEEV replication. Collectively, these findings demonstrate that importin α1 facilitates VEEV capsid nuclear import and contributes to virus-induced cytopathogenicity but is dispensable for nucleocytoplasmic trafficking inhibition and viral replication, further highlighting nuclear transport pathways as attractive targets for host-directed antiviral intervention.

## 1 Introduction

Alphaviruses are single-stranded, enveloped, positive-sense RNA viruses of the *Togaviridae* family that cause significant human and veterinary diseases worldwide. They are divided into two groups based on genetic relatedness, clinical features, and geographic distribution. Old World alphaviruses, including chikungunya virus (CHIKV), Ross River virus, Mayaro virus, and Sindbis virus, are primarily associated with musculoskeletal disease, including severe joint and muscle pain and arthritis, and are endemic to Africa, Asia, Australia, and Europe (Suhrbier et al., 2012). In contrast, New World (NW) alphaviruses, such as Venezuelan, eastern, and western equine encephalitis viruses (VEEV, EEEV, and WEEV, respectively), are highly neurovirulent, causing encephalomyelitis, meningitis, and other severe neurological complications (Carrera et al., 2013; Morens et al., 2019). NW alphaviruses are endemic to the Americas, including the United States (Kumar et al., 2018). The geographic spread of mosquito-borne alphaviruses continues to be shaped by anthropogenic factors such as urbanization and climate change. CHIKV, for example, has continued to spread across the Americas since its introduction in 2013 (de Souza et al., 2024). A notable VEEV outbreak was the 1995 VEEV epidemic in South America, which resulted in more than 75,000 human cases, with 3,000 neurological complication cases and 300 deaths. EEEV cases occur annually in the United States, with case fatality rates approximately 50% (Kim and Diamond, 2023).

VEEV is a zoonotic pathogen responsible for periodic epidemics of febrile illness and severe neurological disease in both equines and humans. Equine mortality rates can reach up to 83% during epizootic outbreaks. Although human mortality is lower (~1%), approximately 14% of patients develop long-term neurological complications, including seizures, confusion, photophobia, and coma (Reyna and Weaver, 2023; Ronca et al., 2016; Woodson et al., 2025; VanderGiessen et al., 2024). Beyond its natural pathogenicity, VEEV is considered a potential biothreat due to its low infectious dose, ease of aerosol transmission, and ability to replicate to high titers. As a result, VEEV is classified as an overlap select agent by both the U. S. Centers for Disease Control and Prevention (CDC) and the U. S. Department of Agriculture (USDA) and is listed as a Category B priority pathogen by the National Institute of Allergy and Infectious Diseases (NIAID) (Woodson et al., 2025). Despite its medical and biodefense significance, no FDA-approved therapeutics or vaccines exist for VEEV infection.

The alphavirus genome is approximately 11.5 kb in length and comprises a 5′ cap, two open reading frames (ORFs), and a poly(A) tail. The first ORF encodes four nonstructural proteins (nsP1-4). The second ORF, expressed under the control of the 26S sub-genomic promoter on the negative strand, encodes the structural proteins (Abu Bakar and Ng, 2018). Following viral entry and fusion, the alphavirus nucleocapsid is released into the cytoplasm, where the nonstructural polyprotein is translated and processed to assemble the viral replication-transcription complex (Yin et al., 2024). This complex synthesizes negative-sense RNA, which serves as a template to produce both genomic and sub-genomic RNAs. The sub-genomic RNA is translated into structural proteins, including the capsid, envelope glycoproteins (E1, E2, E3), and the 6 K/transframe (TF) proteins. Lastly, the capsid proteins combine with viral genomic RNA to form the nucleocapsid, facilitating viral glycoprotein maturation of virion (Lee et al., 1996).

**Figure 1:**
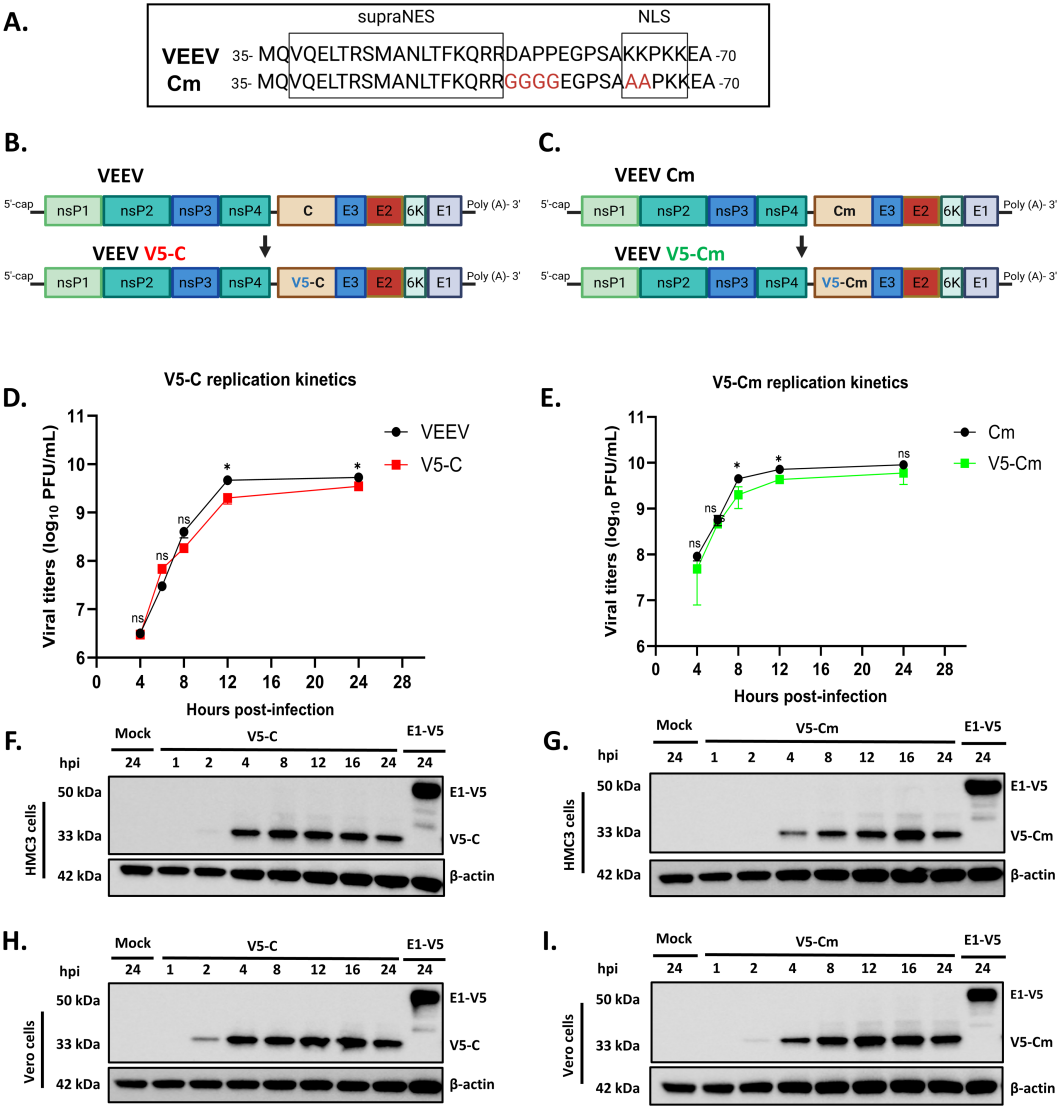
Insertion of V5 tag at the N-terminus of capsid enables successful detection of the capsid. **(A)** Sequence alignment between a region of VEEV TC-83 and VEEV Cm showing key mutation sites [created in BioRender. Kehn-hall, K. (2026), https://BioRender.com/d8zhltb]. **(B,C)** Schematic of VEEV and VEEV Cm with V5 tag inserted at the N-terminus of the capsid protein [created in BioRender. Kehn-hall, K. (2026), https://BioRender.com/nk52hc0]. **(D,E)** Graphs depicting the replication kinetics of V5-C and V5-Cm relative to their respective parental viruses. Vero cells were infected at an MOI 1, and viral titers were determined by plaque assay at 4, 6, 8, 12, and 24 hpi. ^ns^*p >* 0.05, ^*^p ≤ 0.05, ^**^p ≤ 0.01, ^***^p ≤ 0.001, ^****^p ≤ 0.0001. Data are represented as mean ± SD. *N* = 3. **(F–I)** Western blots for V5-C and V5-Cm expression at various time points (MOI 5) in HMC3 and Vero cells. E1-V5 sample was used as a positive control. Mock-infected cells were used as a negative control, and actin was probed as a loading control.

The alphavirus capsid protein consists of two domains with distinct functions. The conserved C-terminal domain possesses autoproteolytic activity, cleaving itself from the structural polyprotein to initiate polypeptide processing, while the highly variable N-terminal domain mediates RNA binding for nucleocapsid formation and virion assembly (Carey et al., 2019). In VEEV, the N-terminal domain also contains nuclear localization (NLS) and nuclear export signals (NES), enabling nucleocytoplasmic shuttling of the capsid (VanderGiessen et al., 2025). Importantly, the capsid antagonizes cellular antiviral response partly through the induction of transcriptional shutoff, and this phenomenon has been mapped to the capsid’s N-terminal domain, independent of its RNA-binding function (Atasheva et al., 2010a; Garmashova et al., 2007b). VEEV capsid is proposed to form a tetrameric complex at the nuclear pore complex (NPC) by simultaneously binding to the host’s import receptors, importin α/β1, and the export receptor (exportin), CRM1, via its NLS and NES, respectively. Unlike typical cellular cargoes, which require RanGTP for CRM1 binding, VEEV capsid contains a supraNES that can stably bind to CRM1 independent of RanGTP (Atasheva et al., 2010a). This capsid-mediated tetrameric complex at the NPC blocks nucleocytoplasmic trafficking of cellular proteins, leading to transcriptional suppression, inhibition of antiviral responses, cytopathic effect (CPE) development, and ultimately cell death (Atasheva et al., 2010a; Garmashova et al., 2007b). Consistently, VEEV capsid mutant (Cm) (Figure 1A), which lacks a functional NLS and cannot block nucleocytoplasmic transport, fails to suppress cellular transcription and antiviral responses, causes less CPE, and exhibits an attenuated phenotype in mice, further highlighting the importance of nucleocytoplasmic blockage for VEEV pathogenicity (Atasheva et al., 2015; Atasheva et al., 2010b).

As part of ongoing efforts to develop effective therapeutics against viruses, targeting host factors important for viral replication has emerged as a promising strategy. Unlike direct inhibition of viral proteins, which often promotes rapid resistance development, targeting host factors essential for viral replication and pathogenicity offers a viable alternative. Nuclear transport receptors, such as importins and exportins, involved in nuclear transport of viral proteins represent key targets. Pharmacological inhibition of CRM1-mediated nuclear export using leptomycin B or selective inhibitors of nuclear export (SINEs) restricts VEEV capsid to the nucleus, preventing its cytoplasmic availability for virion assembly, and thereby reducing viral titers (Lundberg et al., 2013; Lundberg et al., 2016). Similarly, inhibition of importin α/β1-mediated nuclear import with FDA-approved compounds such as ivermectin and mifepristone reduces capsid’s nuclear import and suppresses viral replication (Lundberg et al., 2013). The disruption of capsid-importin α interactions has yielded additional candidate inhibitors, including compounds 1111684, G281-1485 (I0), and G281-1564 (I1), however, the efficacy of these compounds remains limited (Shechter et al., 2017; Thomas et al., 2018). Efforts to develop more potent inhibitors against alphaviruses led to the design of a novel inhibitor I2, through a computational process based on the structure of G281-1485. Computational studies revealed that I2 exhibits stronger binding specificity to importin α1 compared to 1485 and 1564, potentially due to its increased hydrophobicity (Delfing et al., 2023). Additionally, both 1564 and I2 mask the VEEV capsid’s core NLS amino acids to interfere with importin α1-capsid binding (Delfing et al., 2024).

In this study, we characterized the importance of importin α1 using genetic and pharmacological approaches. We show that the complete loss of importin α1 (KPNA2) reduced capsid nuclear import, decreased VEEV-induced cell death, but had no impact on viral replication. In addition, pharmacologically targeting importin α1 using either the next-generation compound I2 or parental compound 1564 (I1) disrupted the interaction between VEEV capsid and importin α1, diminished capsid nuclear import, suppressed VEEV replication, and reduced cell death. Neither loss nor inhibition of importin α1 could relieve the VEEV-induced inhibition of nucleocytoplasmic trafficking. Collectively, our findings demonstrate that importin α1 promotes capsid nuclear localization and contributes to virus-induced cell death while being dispensable for capsid-induced nucleocytoplasmic transport inhibition and viral replication.

## 2 Results

### 2.1 Insertion of a V5 tag at the N-terminus of capsid enables successful detection of the capsid

The study of any protein using molecular and cell biology tools, such as western blot, immunofluorescence, and co-immunoprecipitation, is made possible by the availability of antibodies against the protein of interest. However, in the case of VEEV capsid, there are no antibodies commercially available for this protein. To facilitate these studies, a V5 tag was inserted at the N-terminus of the capsid proteins of both VEEV TC-83 (a BSL-2 strain of VEEV) and VEEV Cm (a VEEV TC-83 strain lacking a functional NLS within the capsid) (Figures 1A–C). The V5 tag is a 14-amino acid epitope tag derived from the P and V proteins of simian virus 5 (Hanke et al., 1992). The VEEV TC-83 molecular clone was used as a template to construct the VEEV TC-83 V5-Capsid tagged molecular clone (VEEV V5-C), while VEEV V5-Cm tagged molecular clone (VEEV V5-Cm) was constructed using a VEEV Cm molecular clone background. The recombinant viruses were produced using a combination of SP6 RNA polymerase-dependent *in vitro* transcription and electroporation. Successful V5 tag insertion was confirmed via Sanger sequencing and western blot. The replication kinetics of the recombinant viruses were compared to their respective parental viruses at various timepoints. The new viral clones replicated productively in Vero cells and exhibited replication kinetics similar to their respective parental strains, indicating that the V5 tag did not interfere with viral replication (Figures 1D,E). The protein expression of both V5-tagged capsid and Cm was determined over time in HMC3 and Vero cells infected at a multiplicity of infection (MOI) of 5. Capsid protein was detected starting at 4 h post-infection (hpi) in HMC3 cells for both VEEV V5-C and VEEV V5-Cm (Figures 1F,G). However, capsid protein was detected as early as 2 hpi in Vero cells (Figures 1H,I). As expected, no expression was detected in the non-infected mock control, and as a positive control, the expression of V5-tagged E1 (E1-V5) glycoprotein was also detected. Furthermore, the stability of the V5 tag inserted in both VEEV V5-C and VEEV V5-Cm was confirmed by passaging both viruses in Vero cells through passage 10. After 10 passaging rounds, V5 signal was detected for both viruses via western blot analysis (data not shown), indicating that the inserted V5 tag is very stable. Moreover, the stability of the V5 tag was further verified through Sanger sequencing, in which all the amplicons derived from the passaged viral clones contained the V5 tag (Supplementary Figure S1). Taken together, these results indicate that V5-tag insertion within the capsid is very stable in both viral clones, and the presence of the epitope tag has a very minimal impact on their replication.

### 2.2 VEEV capsid interacts with both importin α1 and CRM1 in virally infected cells and compounds 1564 and I2 disrupt this interaction

**Figure 2:**
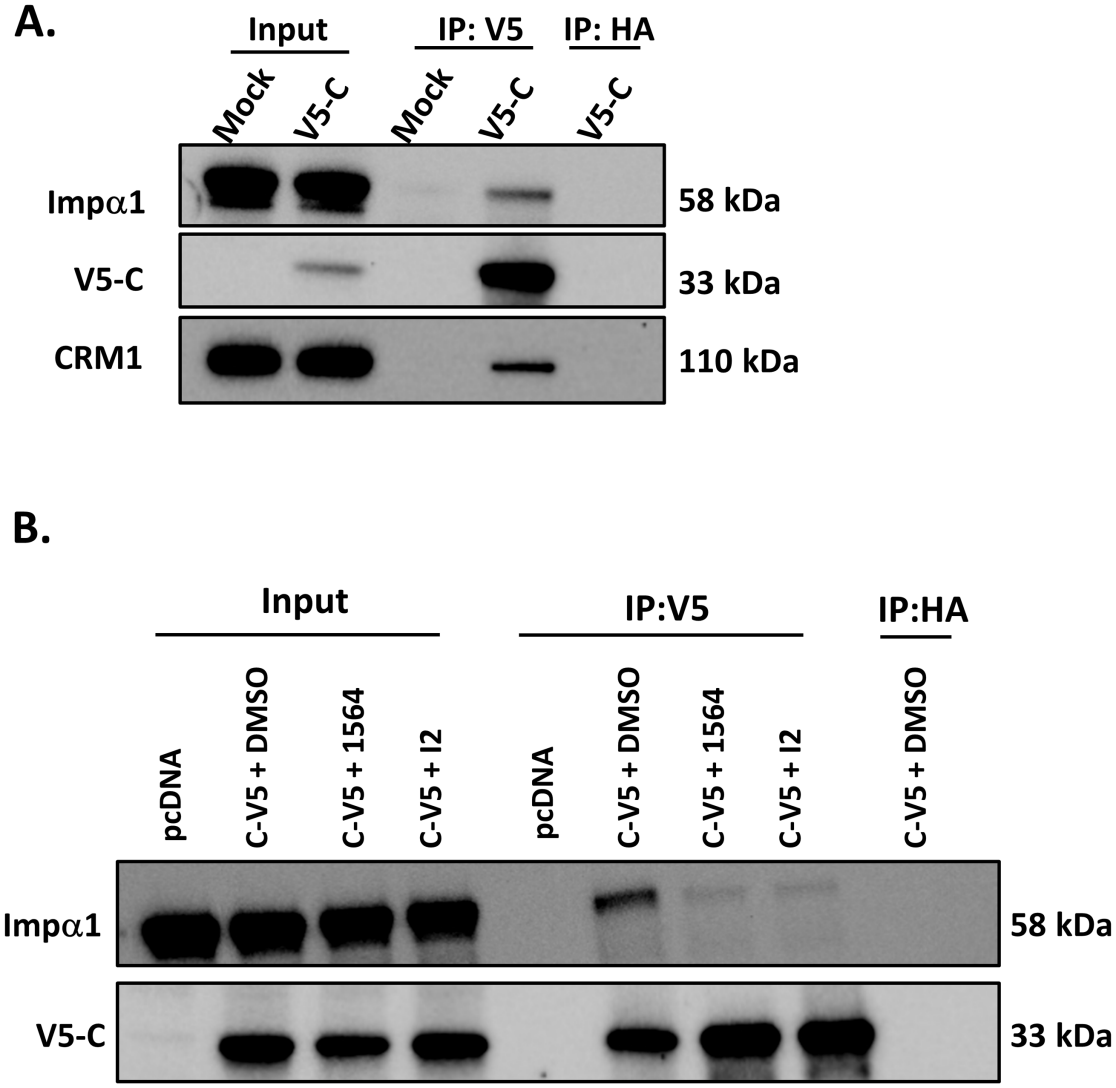
Compounds 1564 and I2 reduce capsid-importin α1 interaction. **(A)** Co-immunoprecipitation was performed using HMC3 cells infected with VEEV V5-C at an MOI of 1. Mock uninfected cells were included as controls. Cells were collected at 9 hpi, protein lysate was quantified and normalized, and subjected to co-immunoprecipitation assay. Pull-down of V5-tagged capsid was done using V5 tag antibody, then western blot was done by probing for importin α1 (KPNA2), CRM1, or V5. A hemagglutinin (HA) antibody pulldown was included as a co-immunoprecipitation control. **(B)** HMC3 cells were pre-treated with respective inhibitor (50 μM) or DMSO for 1 h, then transfected with capsid-V5 plasmid for 6 h, and post-treated with the inhibitor or DMSO for 18 h. Following protein lysates collection and normalization, capsid was pulled down using V5 antibody, then probed for KPNA2 or V5. Pulldown with HA was included as a co-immunoprecipitation control.

In mammalian cells, VEEV disrupts the active transport of cellular macromolecules across the nuclear pore complex (NPC) by interfering with its function. It achieves this partly by binding simultaneously to the host’s import receptors, importin α/β1, and the export receptor CRM1, forming a tetrameric complex that accumulates at the NPC. This complex at the NPC blocks the host’s nucleocytoplasmic trafficking, resulting in inhibition of cellular transcription and antiviral responses, cytopathic effect (CPE) development, and ultimately cell death (Atasheva et al., 2010a; Atasheva et al., 2015; Garmashova et al., 2007b). Although previous studies have reported the interactions between VEEV capsid and nuclear transport receptors, these studies were conducted *in vitro* using biochemical assays (Atasheva et al., 2010a; Shechter et al., 2017; Thomas et al., 2018). To extend this understanding, we investigated the interactions of the full-length VEEV capsid with importin α1 and CRM1 in virally infected mammalian cells. HMC3 (Human microglial clone 3) cell was chosen as a physiologically relevant cell line since VEEV primarily infects glial cells and neurons in the brain (Sharma et al., 2008). HMC3 cells were either mock-infected or infected with VEEV V5-C at an MOI of 1. Cell lysates were collected at 9 hpi, and protein samples were subjected to co-immunoprecipitation. Pulldown of the capsid was done with a V5 antibody, and western blot was performed with importin α1 and CRM1 antibodies. These results demonstrated that both importin α1 and CRM1 interact with VEEV capsid (Figure 2A). A hemagglutinin (HA) antibody pulldown was used as a co-immunoprecipitation control. As expected, neither importin α1 nor CRM1 was detected following HA pulldown, thus confirming that the interactions observed were specific. Taken together, these results represent the first demonstration of the interactions between full-length VEEV capsid with importin α and CRM1 in virally infected cells.

Compounds 1564 and I2 have previously been shown to disrupt capsid and importin α1 interaction *in vitro* via AlphaScreen analysis (Delfing et al., 2024). Next, we aimed to determine the ability of 1564 and I2 (Supplementary Figures S2A,B) to disrupt the interaction between VEEV capsid and importin α1 in virally infected cells. As a first step, the cytotoxicity of each compound in HMC3 cells was assessed. Cells were treated with serial two-fold dilutions of 1564 or I2 for 24 h, after which cell viability was assessed. Both compounds were well tolerated by HMC3 cells, with >80% cell viability recorded at 125 μM (Supplementary Figures S2C,D). Given these results, 50 μM of each compound was selected as a safe concentration for use moving forward. Capsid’s ability to interact with importin α1 was determined in virally infected HMC3 cells treated with DMSO, 1564, or I2. As expected, immunoprecipitation with anti-V5 antibody demonstrated interaction of capsid with importin α1 in VEEV-infected cells treated with DMSO, but not mock cells. Interestingly, both 1564 and I2 significantly interfered with capsid-importin α1 interaction (Supplementary Figures S3A,B). However, these data were confounded by reduced capsid levels in the input samples for 1564 and I2-treated cells. To mitigate this issue, the interaction of capsid with importin α1 was assessed in HMC3 cells transfected with a capsid-V5 expression plasmid and treated with DMSO, 1564, or I2. Capsid was found in complex with importin α1 in HMC3 cells transfected with capsid-V5, but this interaction was markedly reduced in cells treated with compounds 1564 or I2 (Figure 2B). These data demonstrate that compounds 1564 and I2 disrupt capsid-importin α1 interactions.

### 2.3 Knockout of importin α1 reduces capsid nuclear localization

**Figure 3:**
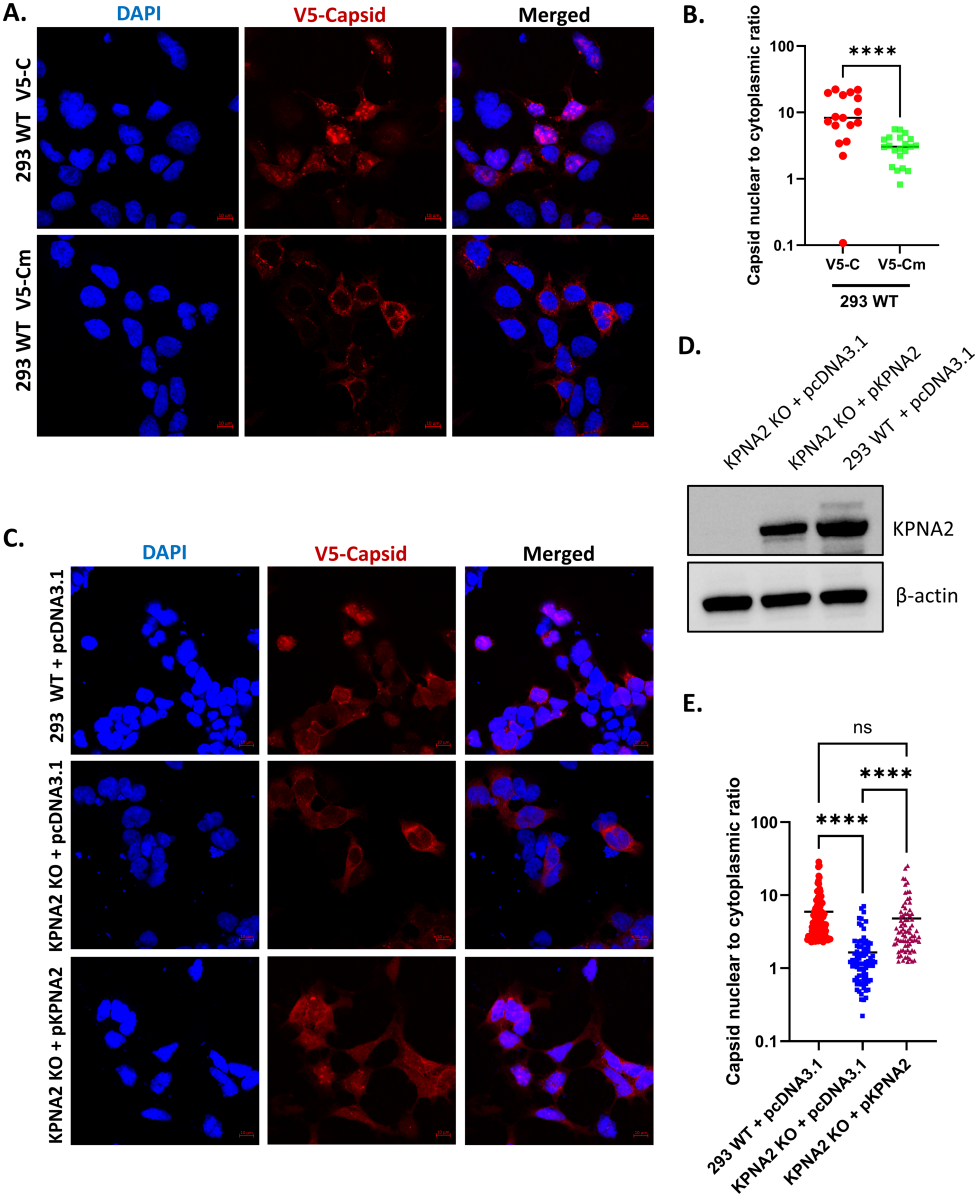
Loss of KPNA2 reduces capsid nuclear localization. **(A)** 293 WT cells on PLL-coated coverslips were infected with VEEV V5-C or VEEV V5-Cm at an MOI of 10 for 9 h, then prepared for immunofluorescence imaging. Images were acquired using confocal microscope. **(B)** The capsid nuclear-to-cytoplasmic ratio in 293 WT cells infected with either VEEV V5-C or V5-Cm was determined using the cellular analysis tool on Gen5 image software. At least 20 cells per condition were utilized in the quantification. Statistics were determined using an unpaired *t*-test. ^ns^*p >* 0.05, ^*^p ≤ 0.05, ^**^p ≤ 0.01, ^***^p ≤ 0.001, ^****^p ≤ 0.0001. **(C)** KPNA2 KO or 293 WT cells on PLL-coated coverslips were transfected with either pcDNA3.1 or KPNA2 plasmid for 24 h, then infected with VEEV V5-C at MOI 10 for 9 h and processed for immunofluorescence microscopy. **(D)** Western blot confirmation for successful transfections of KO cells with pcDNA3.1 or KPNA2 plasmid. **(E)** The capsid nuclear-to-cytoplasmic ratio was determined using the cellular analysis tool on Gen5 mage software. At least 60 cells per condition were utilized in the quantification. ^ns^*p >* 0.05, ^*^p ≤ 0.05, ^**^p ≤ 0.01, ^***^p ≤ 0.001, ^****^p ≤ 0.0001. Slides were stained with DAPI and probed for V5 tag. Blue indicates the nucleus (DAPI) and red indicates V5 tagged capsid protein. Both A, E are presented in log_10_ scale.

The nuclear import of the capsid protein is facilitated by the importin α/β1 pathway and has been shown to play a critical role in viral pathogenesis (Atasheva et al., 2010a; Atasheva et al., 2015; Lundberg et al., 2013). An earlier study by Lundberg et al. (2013) demonstrated the importance of importin α/β1 and CRM1 pathways for the nuclear import and export of VEEV capsid through siRNA-based knockdown experiments. When importin α or both importin α/β1 was knocked down, the nuclear import of the capsid decreased. The effects of siRNA targeting either importin α or both importin α/β1 in reducing the nuclear localization of the capsid were only minimal, arguably because siRNA does not completely prevent protein synthesis. Here, we attempted to test the impact of specifically knocking out importin α1 (KPNA2) on capsid’s localization. For this study, KPNA2 gene knockout HEK293 (KPNA2 KO) cells were generated through CRISPR-Cas9 system. Successful knockout was verified via Sanger sequencing and was confirmed by western blot (Supplementary Figure S4). To validate our assay, the localization of VEEV capsid was assessed in HEK 293 wild-type (293 WT) infected with VEEV V5-C or VEEV V5-Cm as a control. Infection with VEEV V5-C led to the presence of the capsid signal throughout the cell, both in the nucleus and cytoplasm, consistent with previous results (Lundberg et al., 2013; Shechter et al., 2017; Thomas et al., 2018) indicating that the capsid is readily transported in and out of the nucleus in 293 cells (Figure 3A). Conversely, infection with VEEV V5-Cm, lacking a functional NLS, led to predominant cytoplasmic signal of the capsid as expected (Figure 3A). The capsid’s nuclear-to-cytoplasmic ratio was determined, with higher values indicative of predominant nuclear fluorescence and lower values indicating predominant cytoplasmic fluorescence. A significantly higher capsid nuclear-to-cytoplasmic ratio was obtained following the infection of 293 cells with VEEV V5-C, while a lower ratio was noted after VEEV V5-Cm infection (Figure 3B).

Next, to assess the effect of KPNA2 knockout and complementation on capsid localization, KPNA2 KO cells were transfected with either a KPNA2 expression plasmid or pcDNA3.1 control plasmid for 24 h, after which the cells were infected with VEEV V5-C at an MOI of 10 for another 9 h. The 293 WT cells transfected with pcDNA3.1 and infected with VEEV V5-C were included as a control. As expected, 293 WT cells transfected with pcDNA3.1 control plasmid had capsid signal throughout the cell, both in the nucleus and cytoplasm (Figure 3C). Conversely, the loss of KPNA2 in KPNA2 KO cells led to predominant cytoplasmic restriction of the capsid, similar to what was observed following VEEV V5-Cm infection. Importantly, this cytoplasmic restriction of capsid was reversed following the complementation of KPNA2 KO cells with KPNA2 plasmid (Figures 3C,D). Moreover, a high capsid nuclear-to-cytoplasmic ratio was obtained following the infection of 293 WT cells with VEEV V5-C (Figure 3E). This ratio was reduced in KPNA2 KO cells but increased after KPNA2 complementation in KPNA2 KO cells, thus confirming that capsid nuclear localization relies heavily on interaction with KPNA2.

Furthermore, we sought to examine the impact of a complete loss of CRM1 on capsid localization. However, this could not be tested because a knockout of CRM1 was unsuccessful (Supplementary Figure S4). This is partly because of the critical roles CRM1 plays during cellular homeostasis. Moreover, CRM1 knockdown has been reported to lead to cell death, due to the necessity of the protein for cellular development and survival (Callanan et al., 2000). Taken together, these results demonstrate that the complete loss of KPNA2 significantly diminishes the nuclear localization of VEEV capsid, which could be rescued with KPNA2 complementation.

### 2.4 Compounds I2 and 1564 reduce capsid nuclear localization

**Figure 4:**
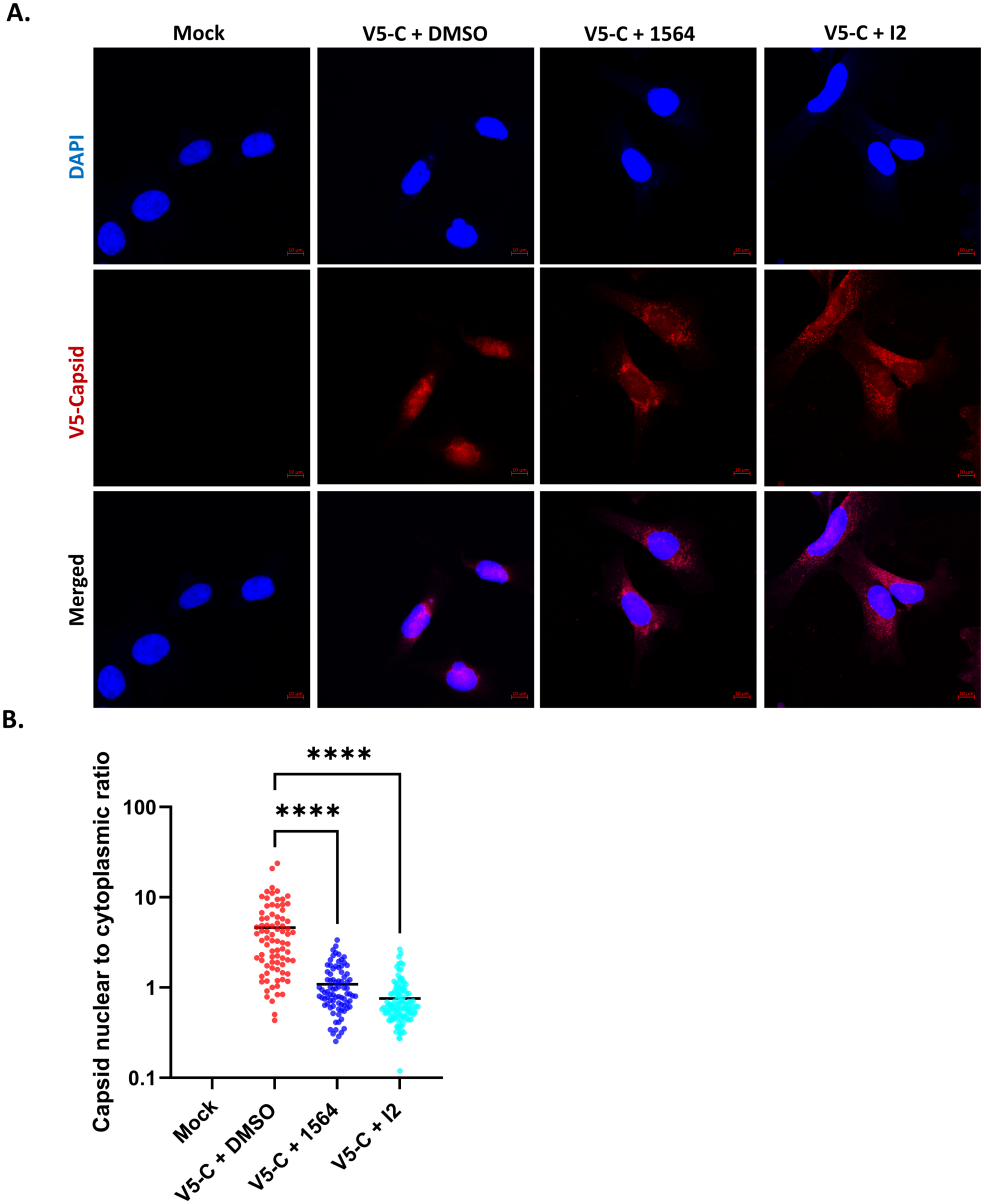
Compounds 1564 and I2 significantly reduce nuclear import of VEEV capsid. **(A)** HMC3 cells on PLL-coated coverslips were pre-treated with 1564 (50 μM), I2 (50 μM), or DMSO, then infected with V5-C at MOI 1, and post-treated with 1564, I2, or DMSO. The slides were then probed, stained, and prepared for immunofluorescence microscopy. Images were acquired using confocal microscope. Blue indicates the nucleus (DAPI) and red indicates V5-tagged capsid protein. **(B)** The capsid nuclear-to-cytoplasmic ratio was determined using the cellular analysis tool on Gen5 Image software. At least 60 cells per condition were utilized in the quantification. Y-axis is in log_10_ scale. ^ns^*p >* 0.05, ^*^p ≤ 0.05, ^**^p ≤ 0.01, ^***^p ≤ 0.001, ^****^p ≤ 0.0001.

Previous data has shown that treatment with compound 1564 led to reduced capsid nuclear localization (Thomas et al., 2018). Here we aimed to extend these data to determine if the next-generation inhibitor I2 was also capable of impacting VEEV capsid nuclear localization. To this end, HMC3 cells were pre-treated with 1564 or I2, then infected with VEEV V5-C, post-treated with the inhibitors for 16 h, and subjected to immunofluorescence processing. Infected cells treated with DMSO were included as controls. Infection with VEEV V5-C led to the distribution of capsid in both the nucleus and cytoplasm within the HMC3 cells (Figure 4A). However, treatment with compound 1564 at a concentration of 50 μM reduced capsid’s nuclear import, with the capsid signal predominantly detected in the cytoplasm (Figures 4A,B). Similarly, compound I2 at a concentration of 50 μM also led to the restriction of VEEV capsid to the cytoplasm (Figures 4A,B). Both inhibitors significantly decreased the capsid nuclear-to-cytoplasmic ratio. A similar phenotype was observed when both inhibitors were tested at 25 μM (Supplementary Figure S5). Taken together, our findings demonstrate that these inhibitors interfere with the shuttling of the capsid to the nucleus.

### 2.5 Loss of importin α1 does not reverse capsid’s block of nucleocytoplasmic trafficking

**Figure 5:**
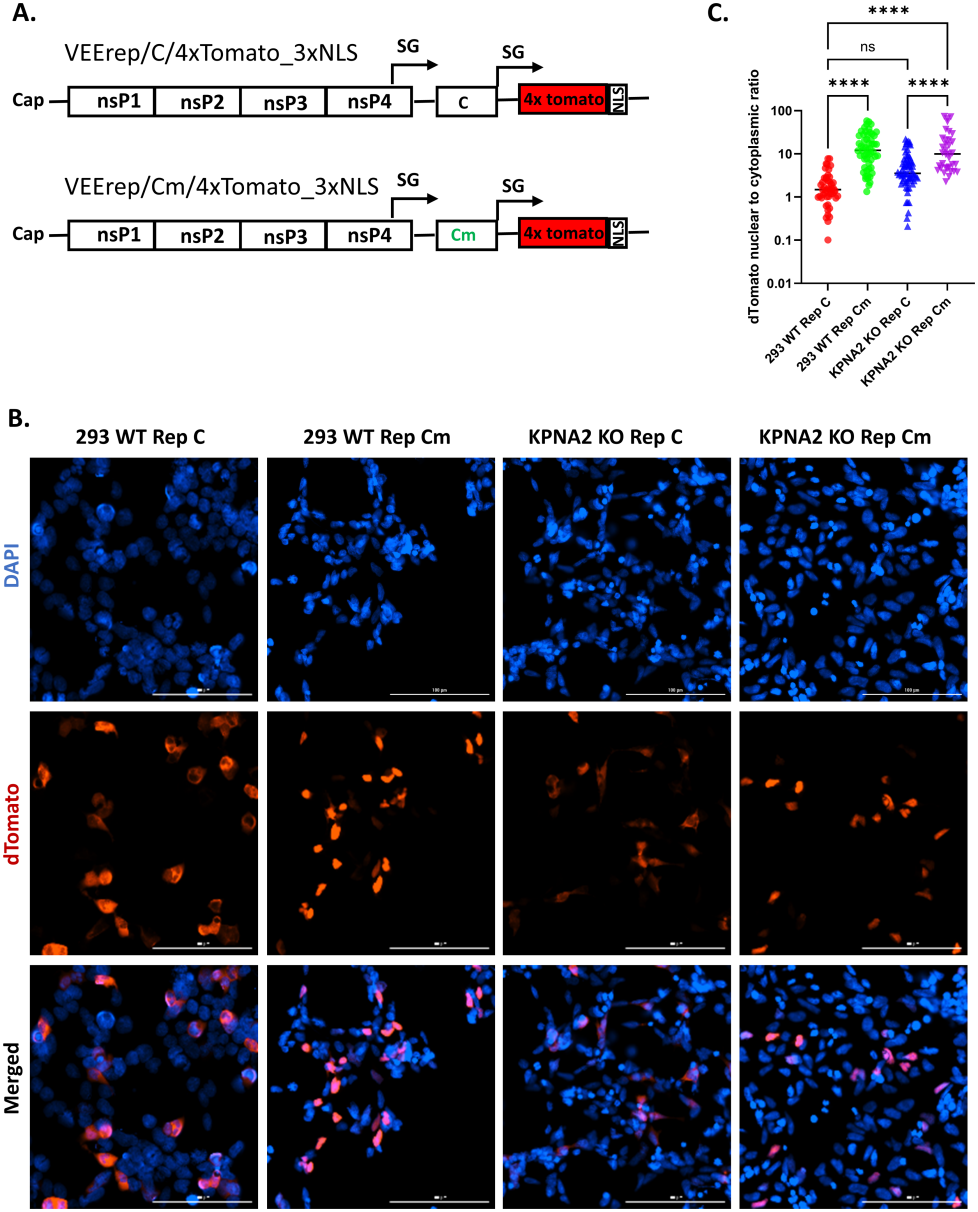
Loss of KPNA2 does not reverse capsid’s block of nucleocytoplasmic trafficking. **(A)** Schematic of the constructs of the two replicon systems (VEErep/C/4xTomato_3xNLS and VEErep/Cm/4xTomato_3xNLS) used for the nucleocytoplasmic trafficking assay. **(B)** 293 WT or KPNA2 KO cells were electroporated with either VEErep/C/4xTomato_3xNLS or VEErep/Cm/4xTomato_3xNLS. At 16 h post electroporation (hpe), cells were prepared for immunofluorescence microscopy. Blue indicates the nucleus (DAPI) and red indicates dTomato. **(C)** The nuclear-to-cytoplasmic ratio of dTomato was determined using the cellular analysis tool on Gen5 Image software. At least 40 cells per condition were utilized in the quantification. Y-axis is in log_10_ scale. ^ns^*p >* 0.05, ^*^p ≤ 0.05, ^**^p ≤ 0.01, ^***^p ≤ 0.001, ^****^p ≤ 0.0001.

Infection of the mammalian cell lines with VEEV TC83 induces rapid shutdown of the cellular transcriptional and translational machinery, which is achieved via inhibition of the host nuclear trafficking by the virus capsid protein (Garmashova et al., 2007b; Atasheva et al., 2008). To test whether the knockout of importin α1 (KPNA2), a key component of the capsid-mediated tetrameric complex, abolishes the nucleocytoplasmic blockage, we utilized VEEV-based replicons modified from those described before (Atasheva et al., 2008) (Figure 5A). In contrast to the original replicons, several modifications were made. Briefly, WT capsid or Cm capsid sequences were placed under the sub-genomic promoter. A second sub-genomic promoter was incorporated, and two double Tomato (dTomato) genes were placed under this promoter. Each dTomato gene encodes a dimer of modified DsRed hence the construct is designated as 4xTomato (or dTomato). The dTomato is a high molecular weight fluorescent protein (~109.5 kDa) that cannot localize to the nucleus by itself. The addition of three copies of SV40 NLS signal (3x NLS) to the dTomato protein allows it to be transported into the nucleus. We developed two types of replicons: one replicon, called Replicon C (VEErep/C/4xTomato_3xNLS), expresses the WT capsid, and the second replicon, called Replicon Cm (VEErep/Cm/4xTomato_3xNLS), expresses capsid with a non-functional NLS and a modified linker located between the NLS and NES (Cm), as earlier described (Atasheva et al., 2010a). After electroporation into 293 WT cells, the replicon transcribes both the capsid and dTomato genes. Consistent with previous results, the presence of the VEEV capsid blocks dTomato entry into the nucleus, making it predominantly accumulated in the cytoplasm (Figure 5B). In contrast, when the replicon expresses Cm protein, dTomato is able to localize to the nucleus, indicating that Cm does not block nucleocytoplasmic trafficking (Figure 5B). This observation is supported by the quantitative analysis which shows that the nuclear-to-cytoplasmic ratio of dTomato signal is higher in the presence of Cm (Figure 5C). To test whether the absence of KPNA2 prevents capsid-induced nucleocytoplasmic blockage, we used KPNA2 KO cells. Knocking out KPNA2 has no significant impact on nucleocytoplasmic blockage, relative to 293 WT with an intact KPNA2 (Figures 5B,C), suggesting that beyond KPNA2 other importin α isoforms may be playing a role in the blockage (Oka and Yoneda, 2018). Moreover, as expected, dTomato significantly localizes to the nucleus in KPNA2 KO cells when Cm is expressed. Thus, altogether our data suggest that while the loss of KPNA2 is sufficient to significantly reduce capsid nuclear localization, it is insufficient to release the nucleocytoplasmic trafficking inhibition.

### 2.6 Compounds 1564 and I2 do not reverse capsid’s block of nucleocytoplasmic trafficking

**Figure 6:**
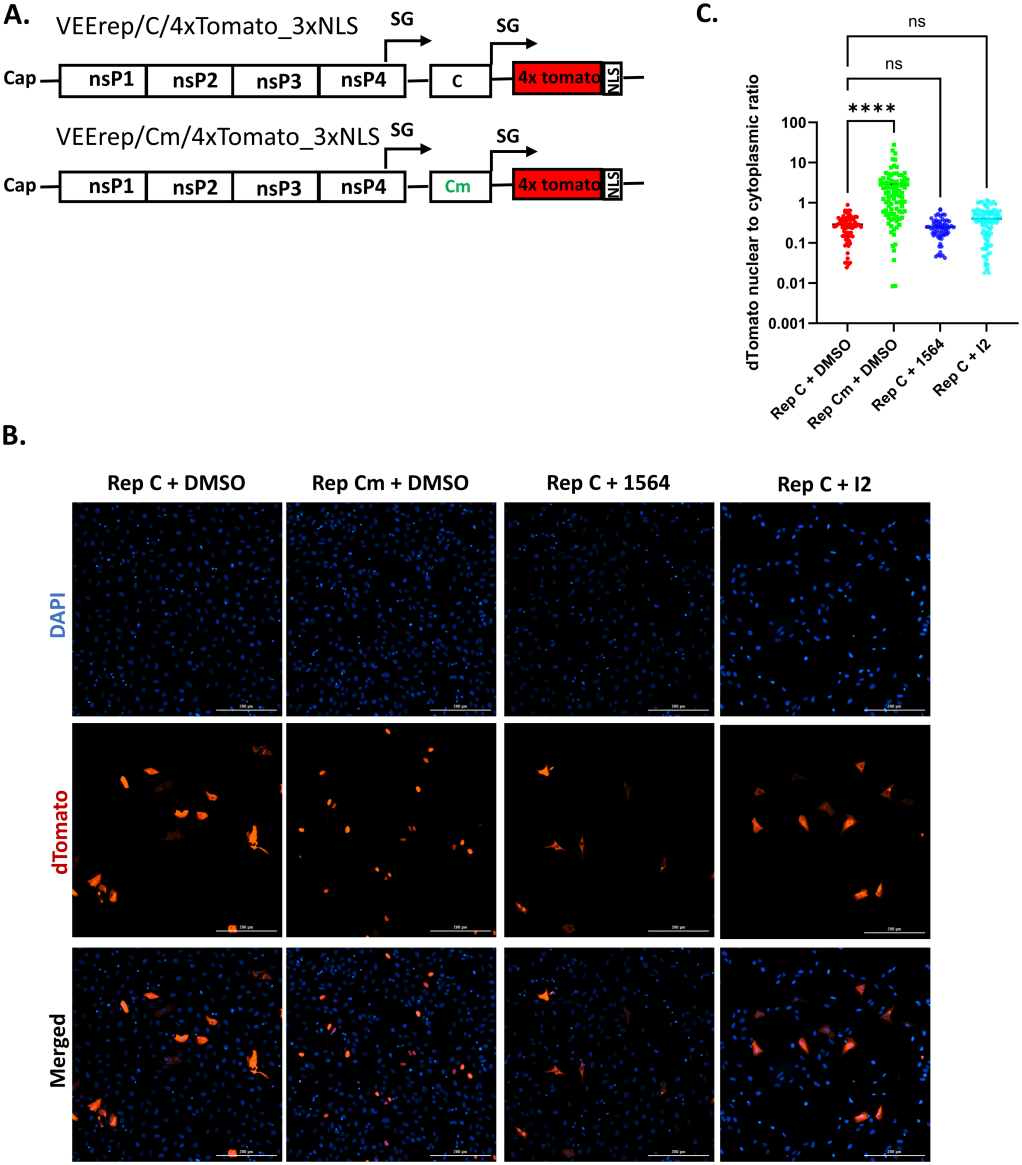
Compounds I2 and 1564 do not reverse capsid’s block of nucleocytoplasmic trafficking. **(A)** Schematic of the VEErep/C/4xTomato_3xNLS replicon and the experimental design. **(B)** Vero cells were pre-treated with 1564, I2 or DMSO for 1 h, after which the cells were electroporated with VEErep/C/4xTomato_3xNLS and post-treated the inhibitors or DMSO for 16 h. Cells were prepared for immunofluorescence microscopy. Blue indicates the nucleus (DAPI) and red indicates dTomato. **(C)** The nuclear-to-cytoplasmic ratio of dTomato was determined using the cellular analysis tool on Gen5 image software. At least 60 cells per condition were utilized in the quantification. Y-axis is in log_10_ scale. ^ns^*p >* 0.05, ^*^p ≤ 0.05, ^**^p ≤ 0.01, ^***^p ≤ 0.001, ^****^p ≤ 0.0001.

Beyond the impact of the inhibitors on the subcellular localization of VEEV capsid, we aimed to assess their impacts on capsid-induced nucleocytoplasmic blockage using the dTomato replicon system. To test this, Vero cells were pre-treated with 50 μM of each inhibitor or DMSO for 1 h, then electroporated with Replicon C, and post-treated with the inhibitor or DMSO for 16 h (Figure 6A). Cells electroporated with replicon Cm or leptomycin B-treated cells electroporated with replicon C were included as controls. As expected, Vero cells electroporated with capsid-expressing replicon have significantly diminished nuclear localization of dTomato. Conversely, cells electroporated with Cm-expressing replicon could not block nucleocytoplasmic transport and significantly increase nuclear localization of dTomato (Figure 6B). Treatment with the CRM1 inhibitor Leptomycin B resulted in dTomato nuclear localization (Supplementary Figure S6). However, both 1564 and I2 compounds at the tested concentration could not reverse the capsid-induced blockage of nucleocytoplasmic trafficking (Figure 6C). Taken together, these results indicate that while both compound 1564 and I2 potently diminish nuclear localization of the capsid, they could not rescue cells from capsid-induced nucleocytoplasmic inhibition.

### 2.7 VEEV capsid interacts with other importin α family members

**Figure 7:**
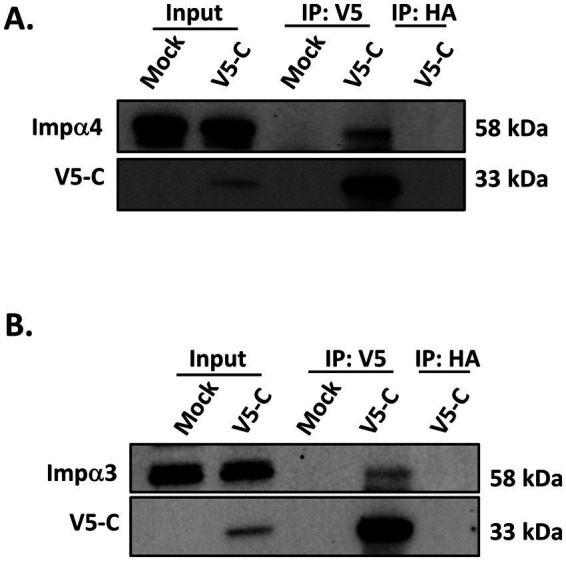
Capsid interacts with other importin α family members. Following infection with VEEV V5-C (MOI 1), HMC3 cells were collected, protein lysates were quantified, and subjected to co-immunoprecipitation assays. A hemagglutinin (HA) antibody pulldown was included as a co-immunoprecipitation control. **(A)** Immunoprecipitation of V5-tagged capsid was done using a V5 tag antibody and the blot was probed with either a KPNA3 (importin α4) antibody or V5 antibody. **(B)** Immunoprecipitation of V5-tagged capsid was done using a V5 tag antibody and the blot was probed with either a KPNA4 (importin α3) or V5 antibody.

Given that knocking out KPNA2 was insufficient to release the capsid mediated nucleocytoplasmic inhibition, we explored the potential that VEEV capsid could interact with other importin α family members and thus compensate for the loss of importin α1. To test this, we performed co-immunoprecipitation assays to determine if VEEV capsid could interact with KPNA3 (importin α4) and/or KPNA4 (importin α3). Importin α4 and importin α3 were selected based on them being targeted by multiple viruses, including Ebola virus, Hendra virus, and Middle Eastern respiratory syndrome coronavirus (MERS-CoV) (Chien et al., 2025; Vogel et al., 2023). Following infection with VEEV V5-C, cell lysates were collected, and protein samples were subjected to co-immunoprecipitation assay. Capsid was pulled down with a V5 antibody, and western blot was performed with either a KPNA3 or KPNA4 antibody. VEEV capsid was found in association with both importin α4 (Figure 7A) and importin α3 (Figure 7B). Taken together, these results show that VEEV capsid also interacts with other importin α isoforms, potentially providing compensatory roles in capsid-mediated nucleocytoplasmic trafficking inhibition following the deletion or disruption of KPNA2.

### 2.8 Treatment with compound I2 or 1564 or loss of KPNA2 partially prevents VEEV-induced cell death

**Figure 8:**
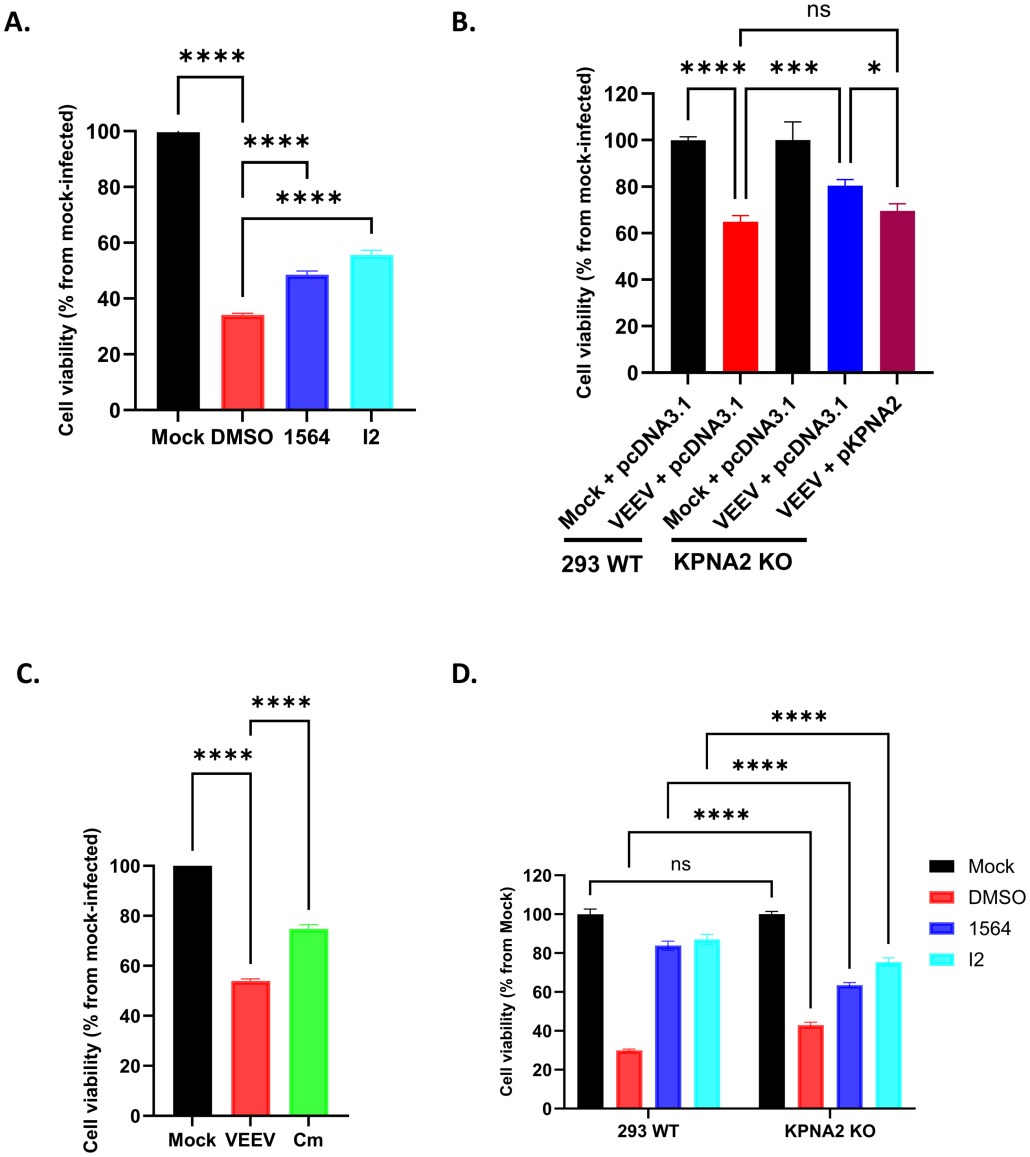
Treatment with I2 or 1564, or loss of KPNA2, partially prevents VEEV induced cell death. **(A)** HMC3 wells were pre-treated with 1564 (50 μM), I2 (50 μM) or DMSO for 1 h. The cells were then infected with VEEV TC-83 at MOI 1 for 1 h, after which they were post-treated with the respective treatment for 24 h. Cell viability was assessed using the CellTiter-Glo assay. Uninfected mock cells were included as a negative control. **(B)** 293 WT or KPNA2 KO cells on poly-L-lysine hydrobromide plates were transfected with either pcDNA3.1 or KPNA2 plasmid. At 24 h post transfection (hpt), the cells were infected with VEEV TC-83 for 24 h. Un-infected mock cells transfected with just pcDNA3.1 plasmid were included as controls. After 24 hpi, cell viability was determined using the CellTiter-Glo assay. **(C)** HMC3 wells were infected with VEEV TC-83 or VEEV Cm at an MOI 1 for 1 h, then fresh media was added. CellTiter-Glo assay was done after 24 h. Uninfected mock cells were included as a negative control. **(D)** 293 WT or KPNA2 KO cells on poly-L-lysine hydrobromide-coated plates were pre-treated with 1564 (50 μM), I2 (50 μM) or DMSO for 1 h. The cells were then infected with VEEV TC-83 at MOI 1 for 1 h, after which they were post-treated with the respective treatment for 26 h. Cell viability was assessed using CellTiter-Glo assay. ^ns^*p* > 0.05, *p ≤ 0.05, ^**^p ≤ 0.01, ^***^p ≤ 0.001, ^****^p ≤ 0.0001.

The N-terminal domain of VEEV capsid, and the NLS-containing region specifically, inhibits cellular transcription and induces the development of cytopathic effects (Atasheva et al., 2015; Garmashova et al., 2007a). Given this, we assessed if the interaction of capsid and importin α1 was essential for VEEV-induced cell death. HMC3 cells were pretreated with 1564, I2, or an equivalent concentration of DMSO for 1 h. Cells were subsequently infected with VEEV at MOI 1, post-treated with the inhibitor or DMSO, and cell viability was measured 24 h later. Infection of HMC3 cells with VEEV caused cell death, leading to a lower than 40% cell viability (Figure 8A). However, this was partly rescued following the treatment of infected cells with 1564 or I2, with the next-generation inhibitor I2 showing better activity. Consistently, lesser cell death was also observed when cells were infected with capsid mutant (Cm) lacking a functional NLS (Figure 8C).

We next utilized KPNA2 KO cells to more specifically determine the importance of importin α1 on VEEV-induced cell death. Relative to mock-infected cells, reduced cell viability was observed following VEEV infection in 293 WT cells. A significant increase in cell viability was observed in VEEV-infected KPNA2 KO cells compared to VEEV-infected 293 WT cells (Figure 8D). Importantly, the complementation of the KPNA2 KO cells with KPNA2 led to reduced cell viability, similar to what was seen in 293 WT cells with an intact *KPNA2* gene, thus confirming KPNA2’s role in VEEV-induced cell death (Figure 8B).

A greater rescue of cell viability was observed with compounds 1564 and I2 as compared to KPNA2 KO (compare Figure 8A with Figure 8B). This suggests that importin α is only partially responsible for the effects observed with 1564 and I2. To directly test this, 293 WT or KPNA2 KO cells were pretreated with 1564, I2, or DMSO control for 1 h. Cells were infected with VEEV at MOI 1, post-treated with the inhibitor or DMSO, and cell viability was measured 26 h later. As expected, a significant increase in cell viability was observed in KPNA2 KO cells infected with VEEV relative to WT cells (Figure 8D). Both 1564 and I2 significantly increased cell viability compared to DMSO treatment in 293 WT cells. We also observed increased cell viability in KPNA2 KO cells treated with 1564 or I2. However, the rescue of cell viability was significantly less than that observed in the 293 WT cells. These results demonstrate that compounds 1564 and I2 not only impact importin α1 but also have other targets that contribute to their impact on cell viability.

Taken together, these results confirm that importin α1 plays a role in VEEV cytopathogenicity and disrupting its function either through inhibitors or KPNA2 KO partially protects cells from VEEV-induced cell death.

### 2.9 Compound 1564 and I2 reduce viral replication in HMC3 cells

**Figure 9:**
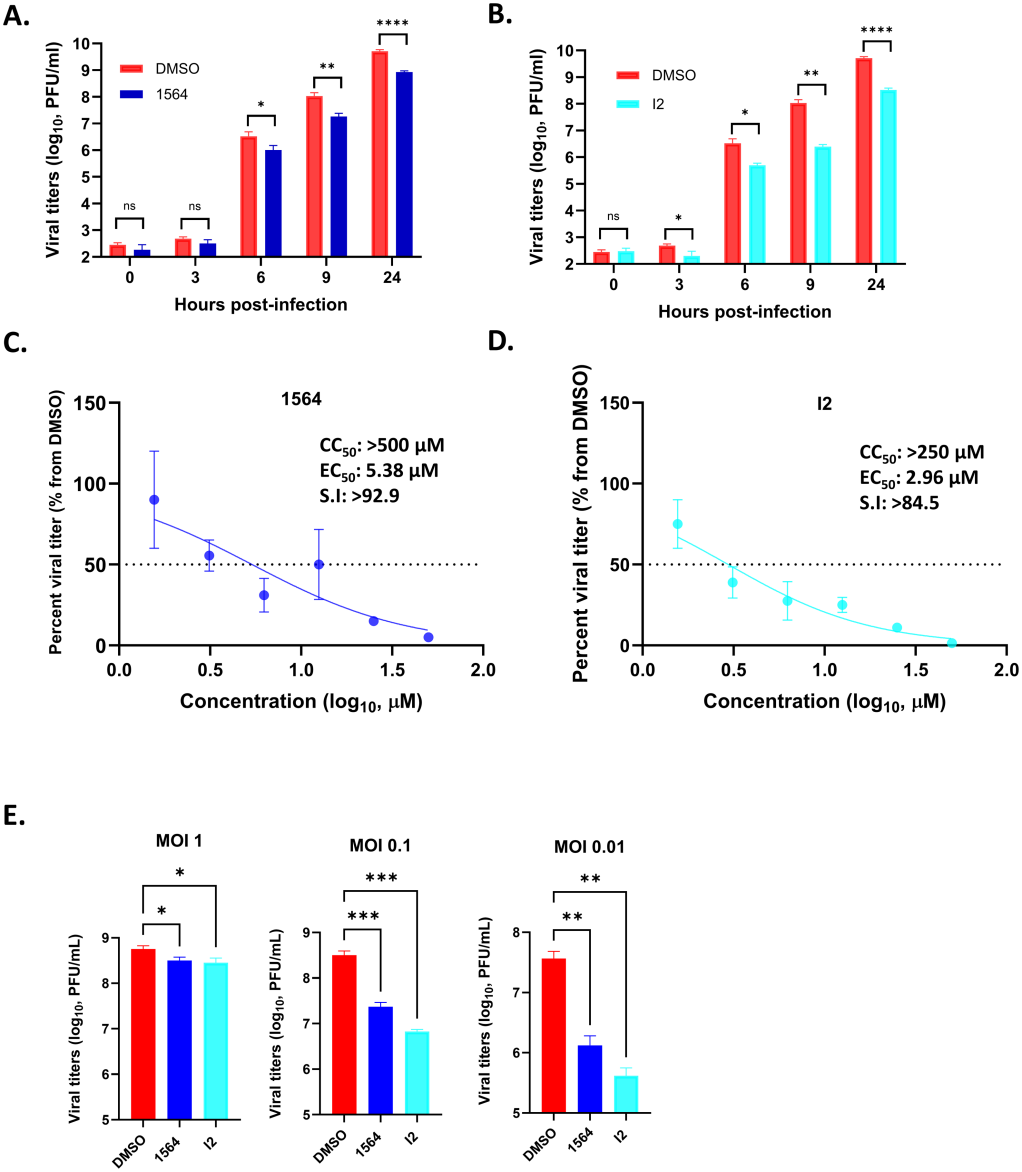
Compound 1564 and I2 reduce viral replication. **(A,B)** Antiviral time-course assays. HMC3 cells were pre-treated with 1564 (50 μM), I2 (50 μM), or an equivalent concentration of DMSO for 1 h, cells were infected with VEEV TC-83 for 1 h, then post-treated with respective inhibitor. After 0, 3, 6, 9, and 24 h, supernatants were collected for plaque assay. **(C,D)** The half-maximal effective concentration (EC_50_) determination. HMC3 cells were pre-treated with the respective inhibitor for 1 h, followed by infection for 1 h, then inhibitor post-treatment. Supernatants were collected at 9 hpi for plaque assay. Selectivity index, the ratio of half-maximal cytotoxic concentration (CC_50_) to EC_50_ ratio, was determined for both inhibitors. **(E)** Multiplicity of infection (MOI)-dependent assay. HMC3 cells were pretreated with 1564 (50 μM), I2 (50 μM), or DMSO, then infected at MOI 1, 0.1, or 0.01, followed by post-treatment with the respective inhibitor. Supernatants were collected at 9 hpi, and viral titers were determined by plaque assay. Asterisks are representative of comparison to DMSO control. ^ns^*p >* 0.05, ^*^p ≤ 0.05, ^**^p ≤ 0.01, ^***^p ≤ 0.001, ^****^p ≤ 0.0001. Data are represented as mean ± SD.

The antiviral activity of both 1564 and I2 was determined against VEEV using HMC3 cells. Cells were pre-treated with non-toxic concentrations of the compounds at 50 μM for 1 h, infected for 1 h at MOI 1, and post-treated with the compounds. Viral supernatants were collected at 0, 3, 6, 9, and 24 hpi, and viral titers were determined using plaque assay. Starting from 3 hpi through 24 hpi, compound 1564 significantly reduces VEEV titers relative to the DMSO control (Figure 9A). A similar, but superior, reduction in viral titers was seen for compound I2. Starting at 3 hpi, I2 reduced viral titers relative to the DMSO control, and this decrease in viral titers continued through 24 hpi. The highest reduction in viral titer of >1.5 log and >1 log was noted at 9 and 24 hpi, respectively (Figure 9B).

Next, the half-maximal effective concentration (EC_50_), i.e., the concentration of the compounds that reduce viral titers by 50%, was determined. 1564 displayed an EC_50_ value of 5.38 μM (Figure 9C), while a lower and better EC_50_ of 2.96 μM was obtained for compound I2 (Figure 9D). The CC_50_ values of 1564 and I2 were calculated to be >500 μM and >250 μM, respectively (Supplementary Figures S2E,F). These results also indicate that relative to its parental compound, G281-1485, which showed very high cytotoxicity (CC_50_ of 55 μM) (Thomas et al., 2018), compound I2 has a better toxicity profile. The selectivity index, which is a measure of the window between toxicity and therapeutic efficacy, was determined to be >92.9 and >84.5 for 1564 and I2, respectively.

Finally, we sought to determine whether there are any differences in the antiviral activity of the inhibitors at different multiplicities of infection (MOIs). Both compounds displayed better activity at lower MOI (MOIs 0.1 and 0.01) (Figure 9E). Taken together, these results indicate that both 1564 and I2 have a good cytotoxicity profile and significantly reduce VEEV titers at different timepoints in HMC3 cells, with I2 demonstrating better antiviral activity.

### 2.10 Loss of importin α1 does not impact VEEV replication kinetics

**Figure 10:**
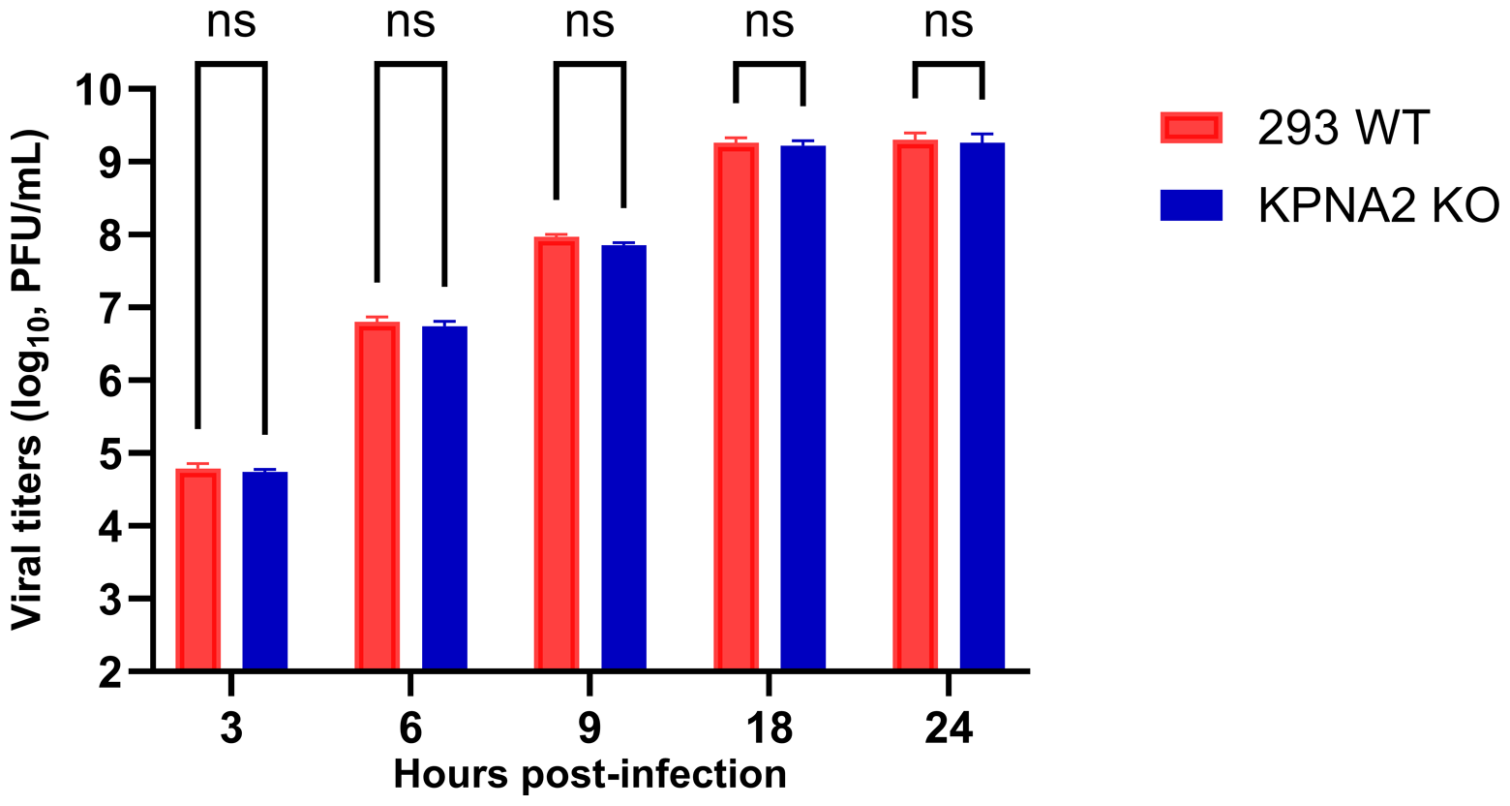
Loss of KPNA2 does not impact VEEV replication kinetics. 293 WT or KPNA2 KO cells on poly-L-lysine hydrobromide-coated plates were infected with VEEV TC83. Supernatants were collected at 3, 6, 9, 18, and 24 hpi, and viral titers were determined by plaque assay. ^ns^*p >* 0.05, ^*^p ≤ 0.05, ^**^p ≤ 0.01, ^***^p ≤ 0.001, ^****^p ≤ 0.0001. Data are represented as mean ± SD.

After showing that the knockout of KPNA2 significantly impacts capsid redistribution, we sought to determine if the lack of KPNA2 has any impact on VEEV replication. To do this, KPNA2 KO or 293 WT cells were infected with VEEV and viral supernatants collected at 3, 6, 9, 18 and 24 hpi. Viral supernatants were subjected to plaque assay. Relative to 293 WT control, the absence of KPNA2 does not impact VEEV replication at all the tested timepoints (Figure 10). These results indicate that while importin α1 is important for capsid localization, it does not directly play a role in VEEV replication.

## 3 Discussion

Nuclear transport receptors, including importins and exportins, have been shown to be exploited by viruses to facilitate replication or evade cellular antiviral responses, making them attractive targets for host-directed antiviral development. While the importance of these receptors is well established for many DNA viruses and RNA viruses with a nuclear replication phase (Resa-Infante et al., 2014), their relevance for cytoplasmic RNA viruses has only started to receive attention. Several RNA viruses, such as Zika virus (ZIKV) and porcine reproductive and respiratory syndrome virus (PRRSV), require importin proteins for their replication (Yang et al., 2018). Moreover, numerous RNA viruses antagonize host innate immune responses by competing with antiviral transcription factors for nuclear transport receptors. Examples include Hepatitis C virus (HCV) NS3/4A (Gagne et al., 2017), Japanese encephalitis virus (JEV) NS5 (Ye et al., 2017), and SARS-CoV-2 ORF6 (Miorin et al., 2020) that antagonize innate immune response through preferential interaction with nuclear import proteins. In this study, we sought to define the role of importin α1 (KPNA2) in VEEV capsid interaction, capsid subcellular localization, nucleocytoplasmic trafficking inhibition, viral replication, and VEEV-induced cell death. To facilitate these studies, we generated recombinant viruses that express capsid protein with a V5 tag. Successful use of tagged recombinant alphaviruses has been reported by other studies (Barrera et al., 2021; Panny et al., 2023). Using this system, we demonstrated that VEEV capsid associates with both importin α1 (KPNA2) and CRM1 in virally infected cells. While a prior work reported the interactions between importin α1/β1 and CRM1 using an H68 fragment derived from the N-terminus of the capsid (Atasheva et al., 2010a), our study is the first to demonstrate these interactions using a full-length capsid in the context of virally infected cells.

Targeting the interaction between VEEV capsid and importin α has been previously explored as an antiviral strategy, although the efficacy of these inhibitors remains limited (Shechter et al., 2017; Thomas et al., 2018). To overcome these limitations, subsequent efforts to develop more potent inhibitors against alphaviruses led to the computational design of the next-generation compound I2. Computational analyses demonstrate that both I2 and the previously identified compound 1564 (I1) bind to importin α1 (Delfing et al., 2023), with compound I2 exhibiting increased hydrophobicity, an attribute that may enhance its ability to cross the blood–brain barrier and improve therapeutic relevance against neuroinvasive alphaviruses. More recent work further revealed that both I2 and 1564 mask the VEEV capsid’s core NLS residues, thereby interfering with capsid-importin α1 binding (Delfing et al., 2024). These findings were supported by AlphaScreen assays that demonstrate disrupted capsid-importin α1 interaction in the presence of the inhibitors. Extending on these findings, our present study provides direct experimental evidence, through co-immunoprecipitation assay, that I2 and 1564 impair capsid’s interaction with importin α1 in both transfected and virally infected cells.

Many viruses exploit importin α receptors to facilitate nuclear localization of viral proteins. For instance, nuclear import of Nipah virus W protein is dependent on importin α and results in the inhibition of Toll-like receptor (TLR) signaling pathway (Shaw et al., 2005). Similarly, VEEV capsid has been shown to rely on the importin α/β1 pathway for nuclear import (Atasheva et al., 2010a; Lundberg et al., 2013); however, limited information is available on the specific importin α isoform mediating this process. In this study, we identify importin α1 (KPNA2) as a key determinant of VEEV nuclear localization. Our data show that complete loss of importin α1 through CRISPR-Cas9 knockout markedly diminished capsid nuclear localization, a phenotype that was rescued upon KPNA2 complementation. Consistent with this genetic approach, pharmacological disruption of importin α1 using either 1564 or I2 similarly impaired nuclear localization of the capsid. Given that EEEV capsid also harbors putative NLS (Aguilar et al., 2008), future studies will investigate whether importin α1 similarly contributes to EEEV capsid nuclear import. In CHIKV, an Old-World alphavirus, KPNA4 (importin α3) has however been identified as the major importin α isoform mediating nuclear import of nsP2 protein (Fros et al., 2013; Thomas et al., 2013), highlighting that alphaviruses may preferentially exploit distinct importin α isoforms to facilitate nuclear trafficking of viral proteins.

**Figure 11:**
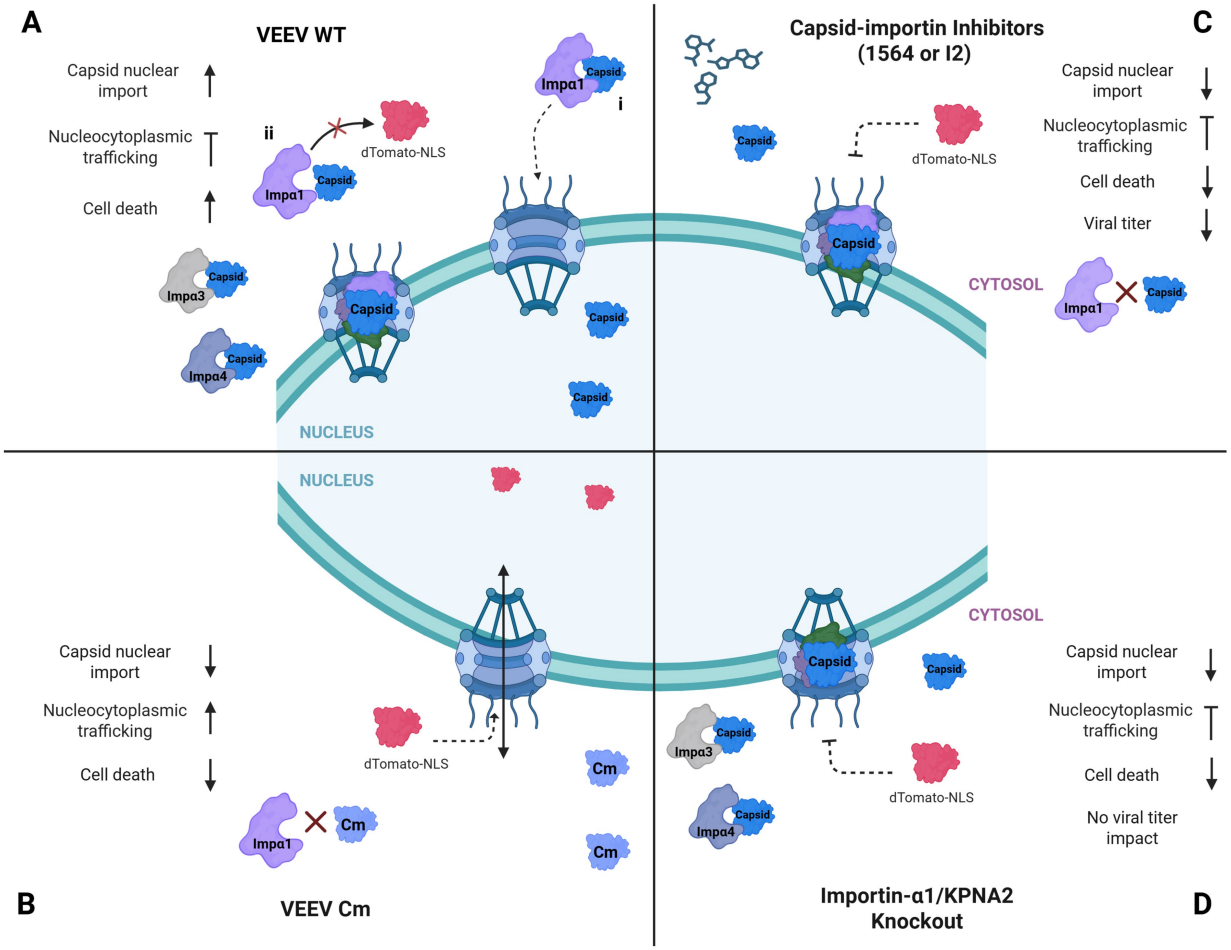
Model for the impacts of 1564, I2, or loss of KPNA2 on VEEV infection. **(A)** We propose a model that following VEEV infection, before nucleocytoplasmic blockage, (i) the capsid is shuttled to the nucleus by importin α1. Later in infection, the capsid is able to block nucleocytoplasmic trafficking by forming a tetrameric complex with CRM1, importin α, and importin β; thus dTomato (a reporter for nuclear import of host proteins) remains cytoplasmic, unable to localize to the nucleus. (ii) An alternative mechanism for inhibition of nucleocytoplasmic trafficking could involve competitive binding of VEEV capsid to importin α to prevent nuclear translocation of other cargo proteins, including dTomato reporter. VEEV capsid can also bind to other importin α isoforms, which may contribute to the inhibition of nucleocytoplasmic trafficking. VEEV infection ultimately results in significant cell death. **(B)** VEEV Cm lacking a functional NLS is unable to localize to the nucleus and does not form a tetrameric complex or compete for importin α1, making it possible for dTomato to shuttle to the nucleus. Ultimately, Cm infection results in less cell death. **(C)** Treatment of VEEV-infected cells with 1564 or I2 reduces nuclear localization of the capsid and rescues cells from VEEV-induced cell death. The treatment also suppressed viral replication. However, inhibitor treatment could not relieve the nucleocytoplasmic trafficking blockage. **(D)** Loss of importin α1 reduces nuclear localization of VEEV capsid and rescues cells from cell death. Importin α1 loss could not relieve the nucleocytoplasmic trafficking blockage possibly because of the interaction of the capsid with other importin α isoforms, which continues to maintain the complex or enable competitive binding. Created in BioRender. Kehn-hall, K. (2026), https://BioRender.com/tifer6k.

VEEV infection in mammalian cells is characterized by a rapid shutdown of host transcriptional and translational machinery, a process mediated by capsid-induced blockage of host nucleocytoplasmic trafficking (Garmashova et al., 2007b; Atasheva et al., 2008). It has been proposed that this blockage results from the formation of a tetrameric complex comprising the capsid, importin α1, importin β1, and CRM1 at the NPC (Atasheva et al., 2010a). Consistent with this model, we observed cytoplasmic retention of dTomato (a reporter for nuclear import of host proteins) in cells electroporated with VEEV replicon expressing the wild-type capsid (Figure 11). Whereas the replicon expressing a capsid mutant (Cm) lacking a functional NLS failed to establish this blockage. Notably, disruption of importin α1, either through loss of importin α1 or pharmacological inhibition, was insufficient to relieve this nucleocytoplasmic blockage (Figure 11). One explanation for this phenomenon may be compensatory contributions from other importin α isoforms, which preserve the block in trafficking even in the absence of importin α1. Mammals encode seven importin α isoforms, several of which have been reported to have redundant or compensatory functions (Huenniger et al., 2010; Oka and Yoneda, 2018). Interestingly, we showed that VEEV capsid was also able to interact with both importin α3 (KPNA4) and importin α4 (KPNA3) in infected cells. These findings demonstrate that, while importin α1 facilitates capsid nuclear localization, capsid-mediated nucleocytoplasmic trafficking inhibition likely involves redundant interactions with multiple importin α isoforms. Future studies will systematically determine the relative contributions and importance of the importin α family members to this process, while also exploring their roles in nuclear pore clogging.

There is an inconsistency between the current working model of the nuclear pore complex being clogged during VEEV infection and VEEV capsid being shuttled into the nucleus. One explanation is that VEEV capsid is shuttled into the nucleus early during infection prior to the nuclear pore being blocked (Figure 11Ai). It has been previously hypothesized that the tetrameric complex is initially formed in the cytoplasm before moving to and ultimately blocking the nuclear pore. Also, the capsid/CRM1 dimer could preassemble on cytoplasmic fibers before clogging the pore following interaction with importin α1/β (Atasheva et al., 2010a). An alternative mechanism could also be that VEEV capsid interacts with importin α family members outside the nuclear pore and sequesters them from binding to other cargo proteins (Figure 11Aii). Other viral proteins have been shown to compete with cellular cargo proteins for binding to importins (Gagne et al., 2017; Ye et al., 2017). However, a detailed time course analysis using live cell imaging could help to determine if either or both models hold true.

The N-terminal domain of VEEV capsid, which contains both NLS and NES, has been shown to inhibit cellular transcription and antiviral responses, ultimately leading to CPE and cell death (Atasheva et al., 2015; Garmashova et al., 2007a). Accordingly, we examined whether impairing importin α1 function alters VEEV-induced cell death. Consistent with previous studies, our data show that VEEV infection dramatically increases cell death. Notably, this phenotype was significantly attenuated following disruption of importin α1 function, either through the genetic loss of importin α1 function or pharmacologically, correlating with reduced nuclear localization of VEEV capsid. These findings identify importin α1 as a host factor that plays a critical role in VEEV-induced cytopathogenicity. Based on these observations, we propose a working model in which VEEV capsid localizes to the nucleus in an importin α1-dependent manner and subsequently interacts with one or more nuclear factor(s) that initiate signaling pathway(s) that ultimately facilitate cell death. Similar mechanisms have been described for other neurotrophic viruses. For instance, nuclear localization of dengue virus capsid induces apoptosis via interaction with DAXX (Death Domain Associated Protein) (Netsawang et al., 2010), while the West Nile virus capsid protein translocate to the nucleus to trigger p53-mediated apoptosis following MDM2 sequestration (Yang et al., 2008). Given that EEEV capsid protein plays a role in CPE development and cell death (Aguilar et al., 2008; Garmashova et al., 2007a), it will be important to determine whether importin α1 loss will similarly modulate EEEV-induced cell death. The ability to rescue cells from virus-induced cell death is particularly significant for neurotrophic viruses in which neuronal loss contributes to neurodegeneration and increased disease severity (Woodson et al., 2025). Our study is the first to demonstrate that importin α1 contributes to VEEV-induced cell death, however, further studies are needed to delineate the mechanisms behind this phenotype.

Finally, although treatment with 1564 or I2 reduced viral titers, the genetic loss of importin α1 did not affect viral replication. One plausible explanation for this discrepancy is that, in addition to targeting importin α1, these inhibitors may have other cellular or viral target(s). However, viral serial passaging in the presence of these inhibitors did not lead to mutation accumulation within the capsid (data not shown), suggesting that the additional effects may be host-directed, for example, other nuclear transport receptors. Consistent with this possibility, inhibition of nuclear transport proteins has been shown to suppress replication of other RNA viruses, including ZIKV and PRRSV (Yang et al., 2018). The fact that the replication defect is absent following importin α1 knockout suggests that importin α1 itself does not directly play a role during the viral replication cycle. This is consistent with a previous study that shows that Cm retained in the cytoplasm does not affect viral replication (Atasheva et al., 2015). Collectively, these results demonstrate that importin α1 facilitates VEEV capsid nuclear localization and contributes to virus-induced cell death while being dispensable for capsid-induced nucleocytoplasmic trafficking blockage and viral replication (Figure 11). Future studies employing multi-omics approaches will be essential to globally characterize changes in cellular genes, pathways, and processes that are modulated by importin α1 disruption, thereby providing deeper insights into the role of importin α1 in viral-induced cytopathogenicity and informing the development of host-directed antiviral strategies.

## 4 Materials and methods

### 4.1 1564 and I2 compound production

For this study, both compounds were synthesized and characterized thereafter as previously described (Delfing et al., 2024).

### 4.2 Cell culture

HMC3 cells (ATCC, CRL-3304) were cultured in Minimum Essential Medium (MEM) supplemented with 10% Fetal Bovine Serum (Avantor, 97068-085), 1% Penicillin/Streptomycin (Corning, 30-002-Cl), and 1% L-Glutamine (Corning, 25-005-Cl). Vero cells (ATCC, CCL-81), and BHK-21 cells (ATCC, CCL-10) were maintained in Dulbecco’s Modified Eagle Medium (DMEM) supplemented with 10% Fetal Bovine Serum (Avantor, 97068-085), 1% Penicillin/Streptomycin (Corning, 30-002-Cl), and 1% L-Glutamine (Corning, 25-005-Cl). All cells were maintained at 37 °C with 5% CO₂. KPNA2 knockout HEK293 (KPNA2 KO) cells were generated through CRISPR-Cas9 system and obtained from EDITGENE (EDJ-KQ3289). Genotype verification was performed at the company site by Sanger sequencing. Knockout efficiency was confirmed by western blot.

### 4.3 Plasmids and viral clone design

The VEEV TC-83 plasmid (Kinney et al., 1993), encoding the VEEV TC-83 genome, was generously provided by Dr. Ilya Frolov (University of Alabama Birmingham) (Atasheva et al., 2015). The VEEV Cm plasmid, which encodes the VEEV Cm genome with a mutated nuclear localization signal (NLS) in the capsid, was generated as described (Lundberg et al., 2016). KPNA2 (importin α1) plasmid (plasmid #26678) was obtained from Addgene, and the KPNA2 ORF with T7 tag was transferred into a p3xFLAG CMV-10 backbone using SpeI-HF (R3133S) and BamHI (R0136S) restriction enzymes to allow for neomycin/geneticin selection in mammalian cells (Kelley et al., 2010). Capsid-V5 plasmid, which expresses VEEV capsid with a C-terminal V5 tag, was designed as previously described (Carey et al., 2018).

The VEEV TC-83 plasmid served as a template to construct the VEEV TC-83 V5-Capsid tagged virus (VEEV V5-C), in which a V5 epitope tag was inserted into the N-terminus of the capsid. Similarly, the VEEV Cm infectious clone was used to create the VEEV V5-Cm tagged virus (VEEV V5-Cm), incorporating a V5 tag at the N-terminus of the capsid mutant. Gene blocks containing the V5-capsid gene block (V5-C gene block) and V5-capsid mutant gene block (V5-Cm gene block) were ordered from Integrated DNA Technologies and transformed separately into competent *Escherichia coli* using the One Shot™ TOP10 kit (Invitrogen, C404010). Large-scale plasmid purification was performed with the Qiagen Plasmid Maxi kit (Qiagen, 12165). To design VEEV V5-C plasmid, a VEEV TC-83 plasmid with an ampicillin resistance marker and the V5-C gene block were digested with the restriction enzymes PspOMI (New England Biolabs, R0653) and AflII (New England Biolabs, R0520). Similarly, VEEV V5-Cm plasmid was constructed by digesting a VEEV Cm plasmid and the V5-Cm gene block with the same restriction enzymes. All the digested DNA samples were separated on a 1% agarose gel, and bands of appropriate sizes were excised, extracted, and purified using the QIAquick Gel Extraction kit (Qiagen, 28704). The resulting fragments from either the VEEV TC-83 plasmid or VEEV Cm plasmid and their respective gene block were then ligated using T4 DNA Ligase (Thermo Fisher Scientific, Cat #15224041), inserting the V5 tag at the N-terminus of the capsid or Cm, after the start codon. The ligated plasmids were transformed separately into *E. coli* using the One Shot™ TOP10 kit and plated on 2x YT agar containing 100 μg/mL ampicillin. Selected colonies were cultured in liquid 2x YT medium with 200 μg/mL ampicillin and incubated overnight at 37 °C in a shaking incubator. Plasmid DNA was extracted using the Qiagen QIAprep Spin Miniprep kit (Qiagen, 27106), and successful insertion of the V5 tag was confirmed by gel electrophoresis and Sanger sequencing. The maps for all constructs are available upon request.

### 4.4 Viral production and replication kinetics

VEEV TC-83 (VEEV), VEEV Cm, VEEV V5-C, and VEEV V5-Cm viral stocks were generated by electroporating BHK-21 cells with *in vitro*-transcribed viral RNA derived from VEEV TC-83, VEEV Cm, VEEV V5-C, and VEEV V5-Cm plasmids, respectively, as described below.

The newly constructed VEEV V5-C or VEEV V5-Cm plasmid (or the parental plasmids, VEEV or VEEV Cm) was linearized with the MluI restriction enzyme (New England Biolabs, R0198S), and linearization was verified by 1% agarose gel electrophoresis. *In vitro* transcription was then performed using Ambion™ SP6 RNA Polymerase (Invitrogen, AM2071) with the linearized plasmid as a template, and RNA synthesis was confirmed on a 1% agarose gel. Approximately 5 × 10^6^ BHK-21 cells were electroporated with the *in vitro*-transcribed RNA. Briefly, cells were washed three times with 1 × PBS (pH 7.4, Gibco, 10-010-023), resuspended in 450 μL PBS, and mixed with RNA before being transferred to an electroporation cuvette (BTX, 45-0135). Electroporation was carried out using the ECM 630 system in exponential decay mode under the following conditions: HV mode, 860 V, 25 μF capacitance, and 950 Ω resistance. After electroporation, cells were incubated at room temperature for 10 min before being transferred to T-75 flasks containing culture medium and incubated at 37 °C with 5% CO₂. Supernatants containing VEEV V5-C or VEEV V5-Cm virus were harvested 24 h post-electroporation and stored at −80 °C. For replication kinetics experiments, Vero (1 × 10^5^ cells/mL) or HMC3 (2 × 10^5^ cells/mL) cells were seeded overnight in 12-well plates. The next day, cells were infected with either VEEV V5-C, VEEV V5-Cm, or the parental viruses (VEEV or VEEV Cm) at MOI 1. Infections were carried out for 1 h at 37 °C, with plates gently rotated every 15 min to ensure uniform viral adsorption. After infection, viral inoculum was removed, cells were washed once with 1x PBS (pH 7.4, Gibco, 10-010-023), and 1 mL of fresh medium was added per well. Supernatants were collected at 4, 6, 8, 12, and 24 hpi and processed for plaque assay.

### 4.5 V5-tag stability assay

Vero cells were seeded in 6-well plates at a density of 4 × 10^5^ cells per well. Cells were infected with either VEEV V5-C or VEEV V5-Cm at a multiplicity of infection (MOI) of 0.1, and culture supernatants were harvested after 24 h. The supernatants were diluted 1:100 in 1 mL DMEM and used to infect fresh Vero cells for an additional 24 h. This process was repeated until passage 10. At every second passage, 1 mL of supernatant was harvested and stored at -80 °C for sequencing, while cell lysates were collected for western blot analysis. At passage 10, the collected supernatants were diluted 10-fold in 1 mL DMEM and used to infect fresh Vero cells. After a 1-h adsorption period, the inoculum was removed, and the cells were covered with a 1:1:1 mixture of 1.5% agarose, 2 × EMEM, and DMEM. Plaques became visible in the overlay after 48 h. Ten individual plaques were isolated using the back end of a P200 pipette tip and transferred into separate tubes containing 500 μL DMEM. Tubes were incubated for 1 h on an end-over-end rotator to dissolve the agarose and release the virus. The resulting plaque suspensions were used to infect fresh Vero cells in 6-well plates and were incubated for 24 h. Cells were lysed in Buffer RLT (Qiagen, Lot No. 175015898) supplemented with 1% β-mercaptoethanol (Millipore Sigma, 444203-205ML), and RNA was extracted using the RNeasy Mini Kit (Qiagen, Cat. No. 74106) according to the manufacturer’s instructions. One hundred (100) nanograms of total RNA were subjected to one-step reverse transcription PCR (RT-PCR) using the SuperScript III One-Step RT-PCR Kit (Invitrogen, Cat. No. 12574-026). Amplicons were sent for Sanger sequencing, which confirmed the presence of the V5 tag in all viral clones, both for VEEV V5-C and VEEV V5-Cm samples. Western blot analysis (described below) was performed on cell lysates collected at passage 10 to verify V5-tag expression. Blots were probed with mouse anti-V5 primary antibody (Bio-Rad, MCA1360) and goat anti-mouse secondary antibody (Invitrogen, 32430).

### 4.6 Cell viability assays

Cells were cultured in a 96-well plate (HMC3 at 2 × 10^4^ cells/well) and incubated overnight. Compounds 1564 and I2 were dissolved in DMSO to generate 20 mM stock solutions. Cells were treated with serial two-fold dilutions of 1564 or I2, starting at 500 μM or 250 μM, respectively. Equivalent concentrations of DMSO were included as negative controls. Following 24 h of incubation, cell viability was assessed by measuring ATP production using the CellTiter-Glo® Luminescent Cell Viability Assay (Promega, G7571). Luminescence was recorded with a Promega GloMax® Discover Microplate Reader. Percent viability was calculated by normalizing the average luminescence values of each treatment group to their corresponding DMSO controls and multiplying by 100. The half-maximal cytotoxic concentration (CC_50_) was determined using GraphPad Prism 10.4.1.

### 4.7 Antiviral assays

For antiviral time-course assays, HMC3 cells (2 × 10^5^ cells/mL) were seeded overnight in 12-well plates. The following day, cells were pretreated with 50 μM of 1564, I2, or an equivalent concentration of DMSO in media for 1 h at 37 °C. The medium was then removed, and cells were infected with 200 μL of VEEV TC-83 at a multiplicity of infection (MOI) of 0.1 for 1 h at 37 °C, with plates gently rotated every 15 min to ensure uniform viral adsorption. After infection, the inoculum was removed, cells were washed twice with 1 × PBS, and post-treated with 1 mL of fresh medium containing 50 μM of 1564, I2, or DMSO. Supernatants were collected at 0, 3, 6, 9, and 24 hpi, and viral titers were determined by plaque assay as described below.

The half-maximal effective concentration (EC_50_) was determined using the same procedure, except cells were pretreated with serial two-fold dilutions of 1564, I2, or DMSO, starting at 50 μM. Infection was performed at an MOI of 0.1, followed by post-treatment with the corresponding compound concentrations. Supernatants were harvested at 9 hpi and processed for plaque assay. For the MOI-dependent assay, cells were pretreated with 50 μM of 1564, I2, or DMSO as described above. Infections were carried out at an MOI of 1, 0.1, or 0.01, followed by post-treatment with the same concentration. Supernatants were collected at 9 hpi, and viral titers were determined by plaque assay.

### 4.8 Virus titration by plaque assay

Crystal violet plaque assays were performed as previously described (Shechter et al., 2017). Vero cells (2 × 10^5^ cells/mL) were seeded overnight in 12-well plates. Supernatants were ten-fold serially diluted in DMEM, 200 μL of each diluted inoculum (typically between 10^−3^ and 10^−8^) was added per well and incubated at 37 °C for 1 h, with gentle rotation every 15 min to ensure even viral adsorption. Following infection, inoculum was removed, and cells were overlaid with 1 mL of a 1:1 mixture containing 2 × EMEM [supplemented with 2% penicillin/streptomycin (Corning, 30-002-Cl), 5% FBS (Avantor, 97068-085), 1% L-glutamine (Corning, 25-005-Cl), 1% MEM non-essential amino acids (Corning, 25-025-Cl), and 1% sodium pyruvate (Corning, 25-000-Cl)] and 0.6% agarose in sterile milliQ water. Plates were incubated at 37 °C for 48 h. Cells were then fixed with 10% formaldehyde for at least 1 h, after which agar plugs were carefully removed using a spatula. The monolayers were stained with 1% crystal violet (Sigma, C0775-100G) in 20% ethanol for 30 min, followed by gentle rinsing with running water. Plaques were counted manually, triplicate samples averaged, and viral titers were calculated (PFU/mL = average plaque count × dilution factor × 5).

### 4.9 Co-immunoprecipitation assay

Cells seeded overnight in T-75 flasks were infected with VEEV V5-C for 1 h. After infection, supernatants were removed, cells were washed once with PBS, trypsinized, and transferred into 10 mL of DMEM in 15 mL Falcon tubes. Cells were pelleted at 3,000 rpm for 3 min and washed twice with cold PBS. Cell pellets were lysed in 300 μL clear lysis buffer (CLB) [50 mM Tris–HCl pH 7.4, 120 mM NaCl, 5 mM EDTA, 0.5% NP-40, 50 mM NaF, 0.2 mM Na3VO4, and EDTA-free complete protease inhibitor cocktail (Roche, 11697498001)], and protein lysates were quantified via Bradford reagent (ThermoFisher, 23236) for co-immunoprecipitation. One mg of protein lysate was incubated overnight at 4 °C with 1 μg of anti-V5 (BioRad, MCA1360) primary antibody. The next day, Dynabeads Protein G (Invitrogen, 10003D) were washed twice with citric phosphate buffer, twice with PBS, and resuspended in 50 μL CLB. Fifty microliters of the beads were added to the co-immunoprecipitation samples and incubated for 60 min at room temperature on a rocker. Beads were then washed twice with 500 μL of TNE 150 buffer (50 mM Tris, pH 7.5, 150 mM NaCl, 1 mM EDTA) containing 0.1% NP-40, followed by two washes with PBS. The final pellet was resuspended in Blue Lysis Buffer [BLB, composed of 25 mL 2x Novex Tris-Glycine SDS Sample Loading Buffer (Invitrogen, LC2676), 20 mL T-PER Tissue Protein Extraction Reagent (ThermoFisher, 78510), 200 μL 0.5 M EDTA (pH 8.0), 3 Complete Protease Inhibitor tablets, 80 μL 0.1 M Na₃VO₄, 400 μL 0.1 M NaF, and 1.3 mL 1 M dithiothreitol (DTT)], boiled at 95 °C, separated by SDS-PAGE, and analyzed by western blot as described below.

### 4.10 Western blot

Cells were lysed in BLB and lysates were boiled for 10 min. Twenty μL per sample was resolved on a NuPAGE 4–12% Bis-Tris gel (Invitrogen, NP0322BOX) and transferred to a methanol-activated PVDF (polyvinylidene difluoride) Transfer Membranes (ThermoFisher, 88518). Transfer was performed on ice for 70 min at 20 mA in 1 × Trans Blot buffer (VWR, 10128–706). Membranes were blocked with 5% milk in 1 × Tris-Buffered Saline solution with 0.1% Tween (TBST) for 1 h at room temperature and incubated overnight at 4 °C with primary antibodies diluted in 0.5% milk/TBST. Mouse anti-V5 (BioRad, MCA1360) and rabbit anti-KPNA2 (Bethyl, A300-484A) were used at 1:10000; rabbit anti-CRM1 (Bethyl, A300-469A) was used at 1:2000; anti-KPNA3 (Bethyl, A301-626A) at 1:500, and anti-KPNA4 (Proteintech, 12463-1-AP) at 1:1000. Following three 5-min washes in 1x TBST and one in 1x Tris-Buffered Saline (TBS), membranes were incubated with secondary antibodies at 1:2000 (goat anti-mouse IgG, Invitrogen 32430; or goat anti-rabbit IgG, Invitrogen 32460) prepared in 0.5% milk/TBST for 1 h at room temperature. After identical washing steps, membranes were developed using the SuperSignal™ West Femto Maximum Sensitivity Substrate (ThermoFisher, 34095) and imaged with a BioRad ChemiDoc™ Imaging system.

### 4.11 Capsid localization assays

To determine the impact of the inhibitors on subcellular capsid localization, HMC3 cells were grown in 6-well plates with poly-L-lysine (PLL)-coated coverslips (Neuvitro corporation, GG-22-1.5-PLL). The cells were pre-treated with 50 μM or 25 μM concentration of 1564, I2, or DMSO. After 1 h incubation, the media was removed, and cells were infected with VEEV V5-C at an MOI of 1 for 1 h at 37 °C. Viral inoculum was removed, and cells were washed twice with 1 × PBS, and post-treated with 1 mL of fresh medium containing 50 μM or 25 μM of 1564, I2, or DMSO for 16 h. The slides were processed for immunofluorescence microscopy as described below.

To assess the effect of KPNA2 knockout on capsid localization, KPNA2 KO or HEK 293 wild-type (293 WT) cells at 2.5 × 10^5^ cells/mL were seeded overnight in 6-well plates with PLL-coated coverslips, then transfected with either pcDNA3.1 or KPNA2 plasmid for 24 h using the Mirus TransIT®-293 transfection reagent (Mirus, MIR 2704), and infected with VEEV V5-C at an MOI of 10 for 9 h. Cells infected with VEEV V5-Cm were included as a control. The slides were then processed for immunofluorescence microscopy as described below.

### 4.12 Immunofluorescence microscopy

Immunofluorescence microscopy was performed as previously described (Panny et al., 2023). Cells grown on PLL-coated coverslips were fixed with 4% paraformaldehyde. The coverslips were probed with mouse anti-V5 primary antibody (1700) and Alexa Fluor 568-conjugated goat anti-mouse secondary antibody at 1:500 (Invitrogen, A11004). Nuclei were counterstained with DAPI (1:1000). Fluorescence images were acquired using either a Zeiss LSM 880 confocal laser scanning microscope or a Cytation 5 imaging reader (BioTek). Using the images acquired on Cytation 5, the nuclear to cytoplasmic (Fn/Fc) ratio of the respective protein, either capsid or dTomato, was determined using the cellular analysis tool on Agilent BioTek Gen5 Image Prime 3.14 software (Galenkamp et al., 2021). Fn and Fc are the integral fluorescence signals of a specific protein within the nuclei and cytoplasm, respectively.

### 4.13 Replicon system development

VEEV TC-83-based replicons were generated using standard molecular cloning techniques. Plasmids containing cDNA of the VEEV replicon and corresponding sequences were kindly provided by Dr. Frolova (Atasheva et al., 2010a). To construct the VEEV C double-tomato replicon (VEEV C_dTomatoNLS), the double-tomato fragment from the original replicon cDNA plasmid (VEErep/4x-Tomato-3xNLS, Atasheva et al., 2010a) was amplified by PCR using the following primers: 5′-TGG TCA CTA GTG TGA GGC CCC TAT AAC TCT CTA CGG C-3′ and 5′-CGA GTT CTA TGT AAG CAG CTT GCC-3′. The amplified fragment was cloned downstream of the capsid gene into the VEEV TC-83 cDNA plasmid between SpeI and MfeI restriction sites, generating the VEEV C_dTomatoNLS plasmid. To generate the VEEV Cm_dTomatoNLS replicon, the VEEV Cm construct was digested with AflII and PspOMI, and the fragment containing the Cm sequence (with mutated NLS and NES) was ligated into the VEEV C_dTomatoNLS plasmid at the same restriction sites. Plasmid construction was confirmed by gel electrophoresis and DNA sequencing.

### 4.14 Replicon system rescue and tomato redistribution assays

Replicon plasmids were linearized and *in vitro* transcribed as described in Section 4.4 above. Vero cells were electroporated with *in vitro*-transcribed replicon RNAs using a 2-mm gap cuvette with a BTX ECM 630 system, as described above. One-tenth of the electroporated cells were seeded into 6-well plates containing pre-placed, PLL-coated coverslips (Neuvitro Corp. Cat. #GG-22-1.5-PLL). At 16 h post-electroporation, cells were fixed with 4% paraformaldehyde and counterstained with DAPI. Coverslips were mounted onto glass slides, and imaging of dTomato redistribution was performed using a Cytation 5 imaging reader (BioTek) with the associated Gen5 Image Prime 3.14 software. To assess the effect of KPNA2 knockout on tomato redistribution, KPNA2 KO cells and HEK 293 wild-type (293 WT) cells were electroporated with either VEEV C_dTomatoNLS or VEEV Cm_dTomatoNLS replicons, as described above. At 16 h post-electroporation, cells were fixed and analyzed for dTomato redistribution.

To evaluate the effect of compounds 1564 and I2 on dTomato redistribution, Vero cells were pretreated with 50 μM of each compound for 1 h prior to electroporation. Following electroporation, cells were post-treated with DMEM containing the same compound concentration and incubated for 16 h. Cells electroporated with replicon Cm or leptomycin B (45 nM) treated cells electroporated with replicon C were included as controls. After incubation, cells were fixed and analyzed for dTomato redistribution as described above.

### 4.15 Impact of KPNA2 KO on viral titer

To assess the impact of KPNA2 knockout on VEEV viral titers, KPNA2 KO cells and HEK 293 wild-type (293 WT) cells were seeded overnight on plates coated with Poly-L-lysine hydrobromide (Sigma-Aldrich, P2636). The cells were infected with VEEV V5-C virus at an MOI of 1 for 1 h at 37 °C, with plates gently rotated every 15 min to ensure uniform viral adsorption. After infection, the inoculum was removed, and fresh medium was added. Supernatants were collected at 3, 6, 9, 18, and 24 hpi, and viral titers were determined by plaque assay.

### 4.16 Cytoprotective assays

Cells were cultured in a 96-well plate (HMC3 at 2 × 10^4^ cells/well) and incubated overnight. Cells were pretreated with 50 μM of 1564, I2, or an equivalent concentration of DMSO in media for 1 h at 37 °C. The medium was then removed, and cells were infected with 100 μL of VEEV TC-83 at an MOI of 1 for 1 h at 37 °C. After infection, the inoculum was removed and cells were post-treated with fresh medium containing 50 μM or 25 μM of 1564, I2, or DMSO, respectively. Following 24 h incubation, cell viability was assessed by measuring ATP production using the CellTiter-Glo® Luminescent Cell Viability Assay (Promega, G7571). Luminescence was recorded with a Promega GloMax® Discover Microplate Reader. Viability was calculated by normalizing the average luminescence values of each treatment group to the un-infected (mock) control and the cytoprotective effect was determined.

The same procedure was followed to determine the impact of KPNA2 KO on VEEV-induced cell death, but in this case, KPNA2 KO or 293 WT cells were used. Cells at 2.5 × 10^5^ cells/mL were grown overnight in 12 well plates coated with Poly-L-lysine hydrobromide (Sigma-Aldrich, P2636), then transfected with either pcDNA3.1 or KPNA2 plasmid using the Mirus TransIT®-293 transfection reagent (Mirus, MIR 2704). At 24 h post transfection (hpt), the cells were infected with VEEV at an MOI of 1 for 24 h. Un-infected mock cells transfected with just pcDNA3.1 plasmid were included as controls. After 24 hpi, cell viability was determined using the CellTiter-Glo® Luminescent Cell Viability Assay. To determine the impact of the inhibitors on cell viability in KPNA2 KO cells, 293 WT or KPNA2 KO cells on Poly-L-lysine hydrobromide-coated plates were pre-treated with 1564 (50 μM), I2 (50 μM) or DMSO for 1 h. The cells were then infected with VEEV TC-83 at MOI 1 for 1 h, after which they were post-treated with the respective treatment for 26 h. Cell viability was then assessed using CellTiter-Glo Assay.

### 4.17 Statistical analysis

Statistical analysis was performed using GraphPad Prism 10.4.1 software. All experiments were done with at least 3 biological replicates. Unless otherwise noted, statistical analysis was done using One-way ANOVA followed by Tukey’s multiple comparisons test. ^ns^*p* > 0.05, ^*^p ≤ 0.05, ^**^p ≤ 0.01, ^***^p ≤ 0.001, ^****^p ≤ 0.0001.

## Data Availability

The raw data supporting the conclusions of this article will be made available by the authors, without undue reservation.
