# Supplementary material for: Importin α1 contributes to Venezuelan equine encephalitis virus-induced cell death, but is not required for the capsid-mediated blockage of nucleocytoplasmic trafficking
Source: Front Microbiol. 2026 Mar 25;17:1784611. doi: 10.3389/fmicb.2026.1784611 (PMC13057339; doi:10.3389/fmicb.2026.1784611)
Supplement: Supplementary file 1 [file Presentation_1.pptx]

## Slide 1
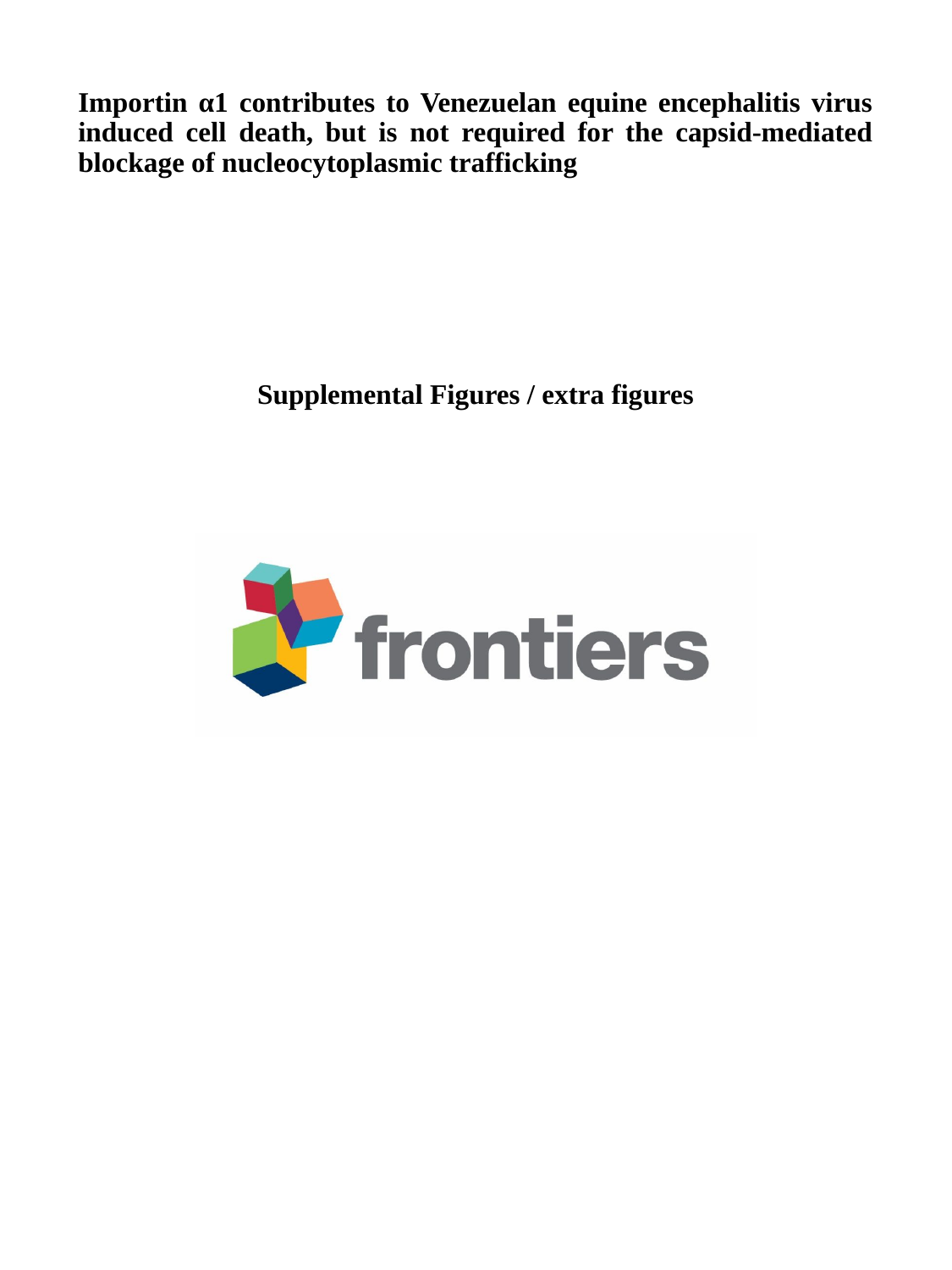

Importin α1 contributes to Venezuelan equine encephalitis virus induced cell death, but is not required for the capsid-mediated blockage of nucleocytoplasmic trafficking
# Supplemental Figures / extra figures

## Slide 2
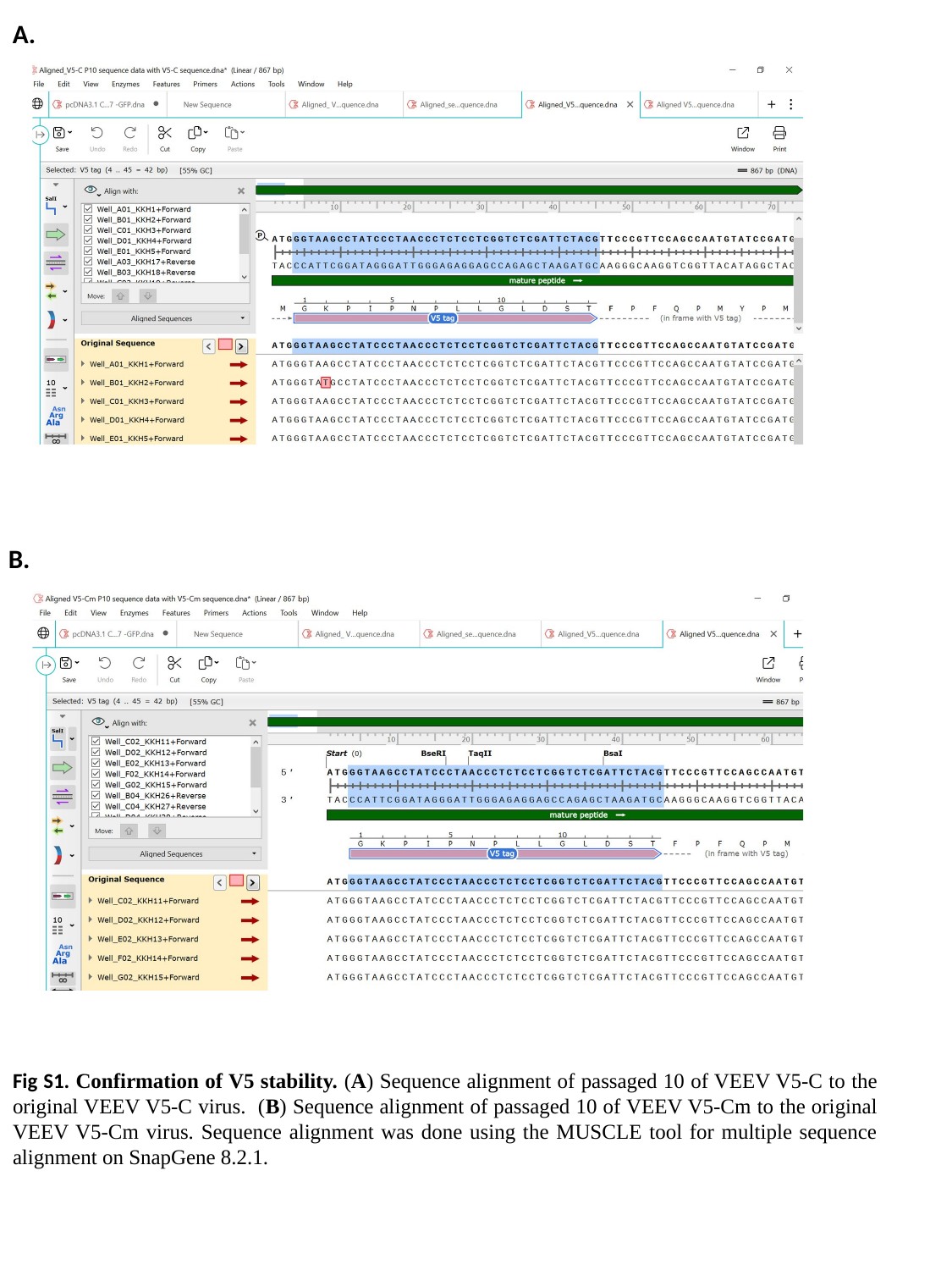

A.
B.
Fig S1. Confirmation of V5 stability. (A) Sequence alignment of passaged 10 of VEEV V5-C to the original VEEV V5-C virus. (B) Sequence alignment of passaged 10 of VEEV V5-Cm to the original VEEV V5-Cm virus. Sequence alignment was done using the MUSCLE tool for multiple sequence alignment on SnapGene 8.2.1.

## Slide 3
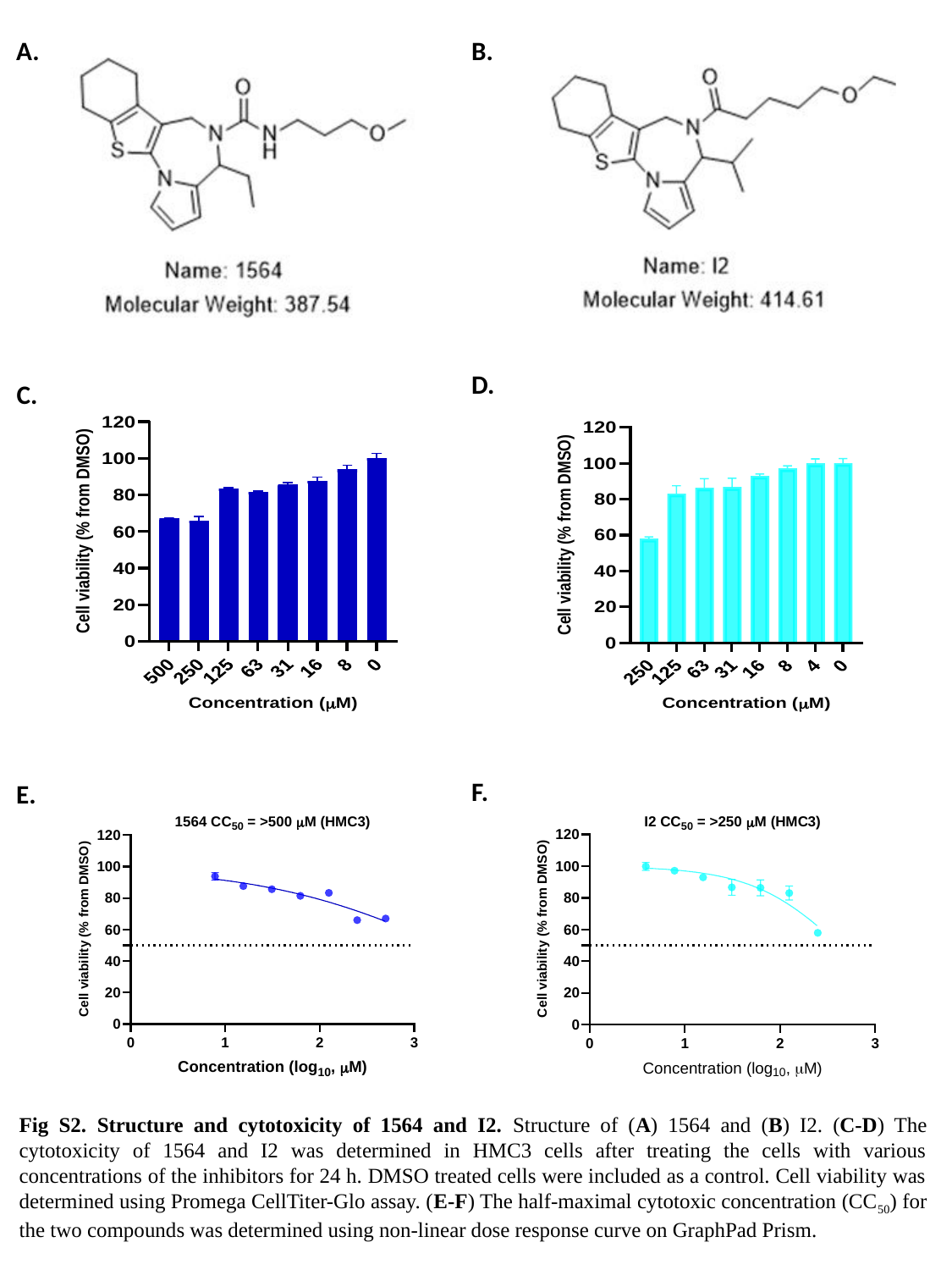

A.
B.
D.
C.
F.
E.
Fig S2. Structure and cytotoxicity of 1564 and I2. Structure of (A) 1564 and (B) I2. (C-D) The cytotoxicity of 1564 and I2 was determined in HMC3 cells after treating the cells with various concentrations of the inhibitors for 24 h. DMSO treated cells were included as a control. Cell viability was determined using Promega CellTiter-Glo assay. (E-F) The half-maximal cytotoxic concentration (CC50) for the two compounds was determined using non-linear dose response curve on GraphPad Prism.

## Slide 4
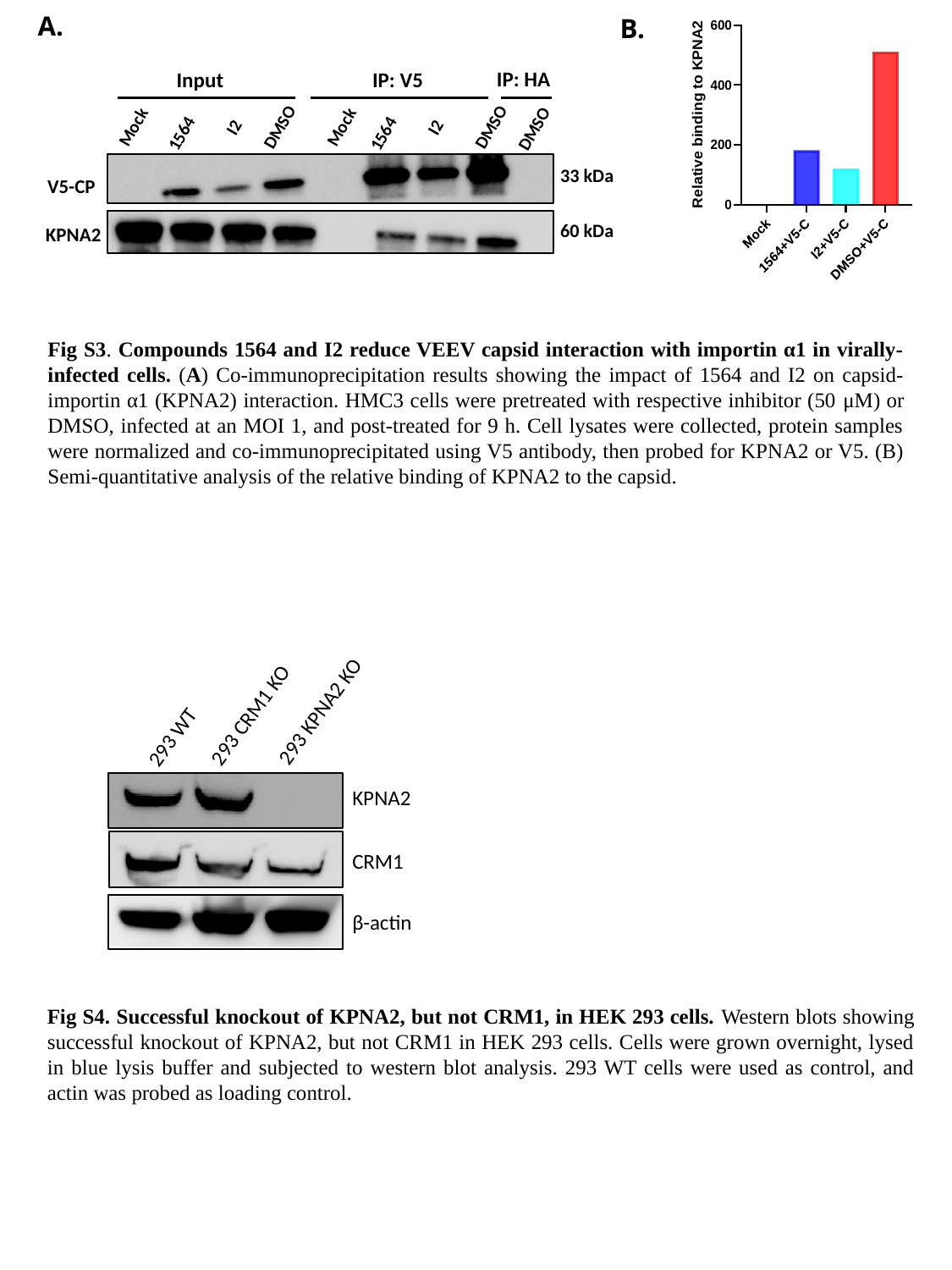

A.
B.
IP: HA
Input
IP: V5
V5-CP
Mock
1564
I2
DMSO
Mock
1564
I2
DMSO
DMSO
33 kDa
60 kDa
KPNA2
Fig S3. Compounds 1564 and I2 reduce VEEV capsid interaction with importin α1 in virally-infected cells. (A) Co-immunoprecipitation results showing the impact of 1564 and I2 on capsid-importin α1 (KPNA2) interaction. HMC3 cells were pretreated with respective inhibitor (50 μM) or DMSO, infected at an MOI 1, and post-treated for 9 h. Cell lysates were collected, protein samples were normalized and co-immunoprecipitated using V5 antibody, then probed for KPNA2 or V5. (B) Semi-quantitative analysis of the relative binding of KPNA2 to the capsid.
293 KPNA2 KO
293 CRM1 KO
293 WT
KPNA2
CRM1
β-actin
Fig S4. Successful knockout of KPNA2, but not CRM1, in HEK 293 cells. Western blots showing successful knockout of KPNA2, but not CRM1 in HEK 293 cells. Cells were grown overnight, lysed in blue lysis buffer and subjected to western blot analysis. 293 WT cells were used as control, and actin was probed as loading control.

## Slide 5
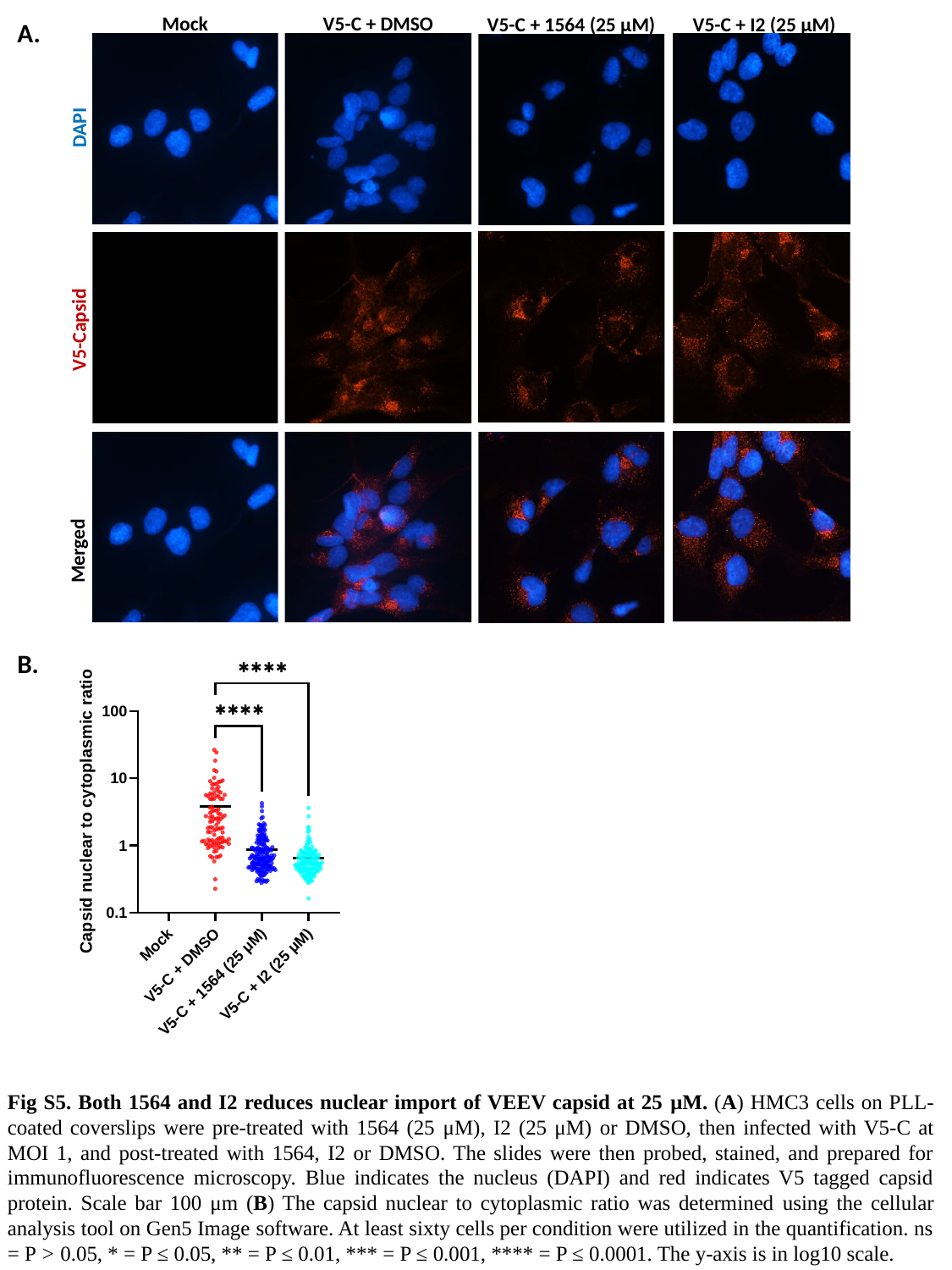

Mock
V5-C + DMSO
V5-C + 1564 (25 µM)
V5-C + I2 (25 µM)
A.
DAPI
V5-Capsid
Merged
B.
Fig S5. Both 1564 and I2 reduces nuclear import of VEEV capsid at 25 μM. (A) HMC3 cells on PLL-coated coverslips were pre-treated with 1564 (25 μM), I2 (25 μM) or DMSO, then infected with V5-C at MOI 1, and post-treated with 1564, I2 or DMSO. The slides were then probed, stained, and prepared for immunofluorescence microscopy. Blue indicates the nucleus (DAPI) and red indicates V5 tagged capsid protein. Scale bar 100 μm (B) The capsid nuclear to cytoplasmic ratio was determined using the cellular analysis tool on Gen5 Image software. At least sixty cells per condition were utilized in the quantification. ns = P > 0.05, * = P ≤ 0.05, ** = P ≤ 0.01, *** = P ≤ 0.001, **** = P ≤ 0.0001. The y-axis is in log10 scale.

## Slide 6
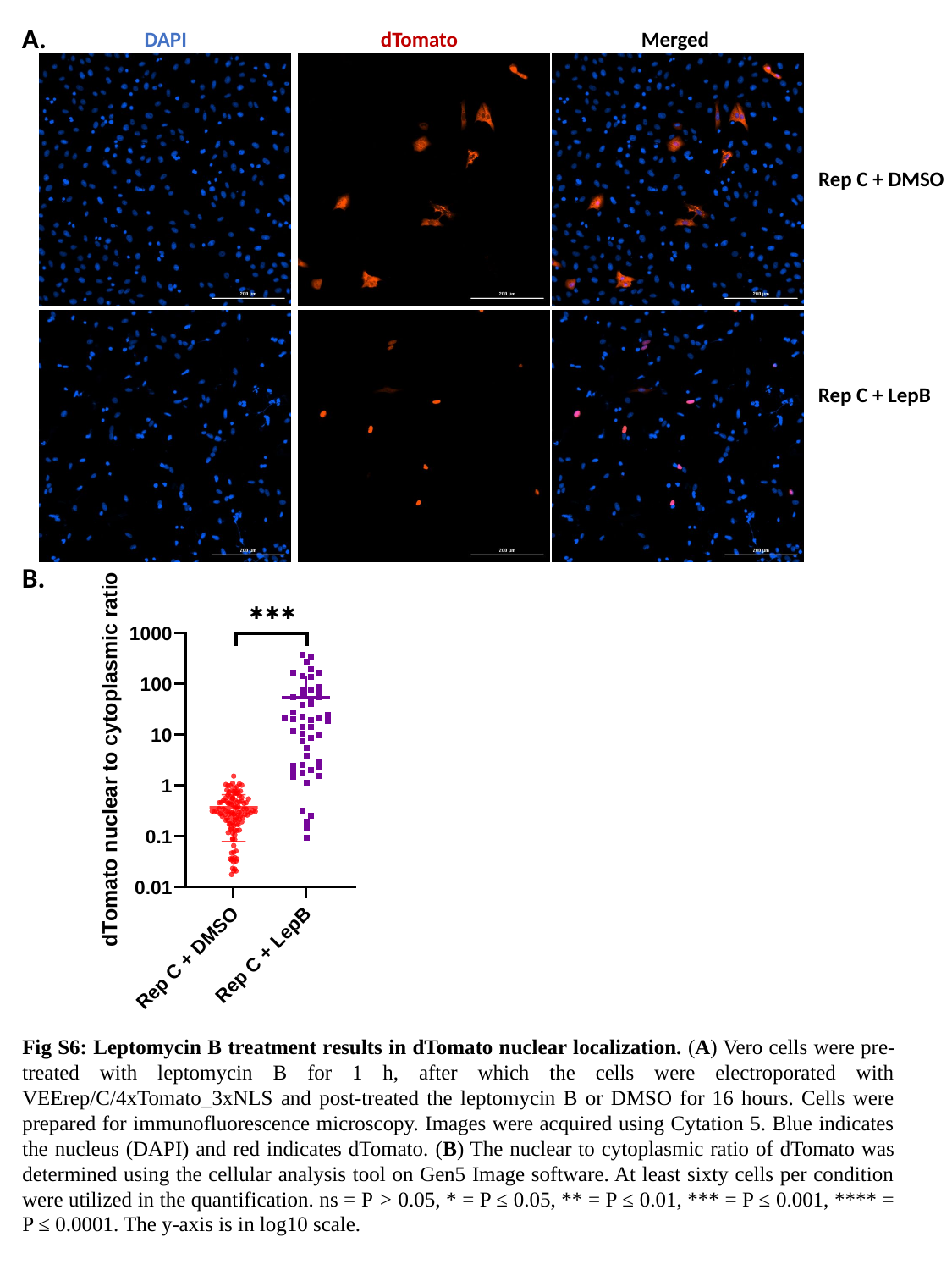

A.
DAPI
dTomato
Merged
Rep C + DMSO
Rep C + LepB
B.
Fig S6: Leptomycin B treatment results in dTomato nuclear localization. (A) Vero cells were pre-treated with leptomycin B for 1 h, after which the cells were electroporated with VEErep/C/4xTomato_3xNLS and post-treated the leptomycin B or DMSO for 16 hours. Cells were prepared for immunofluorescence microscopy. Images were acquired using Cytation 5. Blue indicates the nucleus (DAPI) and red indicates dTomato. (B) The nuclear to cytoplasmic ratio of dTomato was determined using the cellular analysis tool on Gen5 Image software. At least sixty cells per condition were utilized in the quantification. ns = P > 0.05, * = P ≤ 0.05, ** = P ≤ 0.01, *** = P ≤ 0.001, **** = P ≤ 0.0001. The y-axis is in log10 scale.
